# Modulating the polyamine/hypusine axis controls generation of CD8^+^ tissue-resident memory T cells

**DOI:** 10.1172/jci.insight.169308

**Published:** 2023-09-22

**Authors:** Aya G. Elmarsafawi, Rebecca S. Hesterberg, Mario R. Fernandez, Chunying Yang, Lancia N.F. Darville, Min Liu, John M. Koomen, Otto Phanstiel, Reginald Atkins, John E. Mullinax, Shari A. Pilon-Thomas, Frederick L. Locke, Pearlie K. Epling-Burnette, John L. Cleveland

**Affiliations:** 1 Department of Molecular Medicine, Morsani College of Medicine, University of South Florida, Tampa, Florida, USA.; 2 Department of Tumor Biology and; 3 Department of Immunology, H. Lee Moffitt Cancer Center & Research Institute, Tampa, Florida, USA.; 4 Cancer Biology PhD Program, University of South Florida, Tampa, Florida, USA.; 5 Proteomics and Metabolomics Core, H. Lee Moffitt Cancer Center & Research Institute, Tampa, Florida, USA.; 6 Department of Medical Education, University of Central Florida College of Medicine, Orlando, Florida, USA.; 7 Department of Clinical Science,; 8 Sarcoma Department, and; 9 Department of Blood and Marrow Transplant and Cellular Immunotherapy, H. Lee Moffitt Cancer Center & Research Institute, Tampa, Florida, USA.; 10 Precision Medicine Oncology, AbbVie Inc., North Chicago, Illinois, USA.

**Keywords:** Immunology, Metabolism, Polyamines, T cells

## Abstract

Glutaminolysis is a hallmark of the activation and metabolic reprogramming of T cells. Isotopic tracer analyses of antigen-activated effector CD8^+^ T cells revealed that glutamine is the principal carbon source for the biosynthesis of polyamines putrescine, spermidine, and spermine. These metabolites play critical roles in activation-induced T cell proliferation, as well as for the production of hypusine, which is derived from spermidine and is covalently linked to the translation elongation factor eukaryotic translation initiation factor 5A (eIF5A). Here, we demonstrated that the glutamine/polyamine/hypusine axis controlled the expression of CD69, an important regulator of tissue-resident memory T cells (Trm). Inhibition of this circuit augmented the development of Trm cells ex vivo and in vivo in the BM, a well-established niche for Trm cells. Furthermore, blocking the polyamine/hypusine axis augmented CD69 expression as well as IFN-γ and TNF-α production in (a) human CD8^+^ T cells from peripheral blood and sarcoma tumor infiltrating lymphocytes and (b) human CD8^+^ CAR-T cells. Collectively, these findings support the notion that the polyamine-hypusine circuit can be exploited to modulate Trm cells for therapeutic benefit.

## Graphical abstract

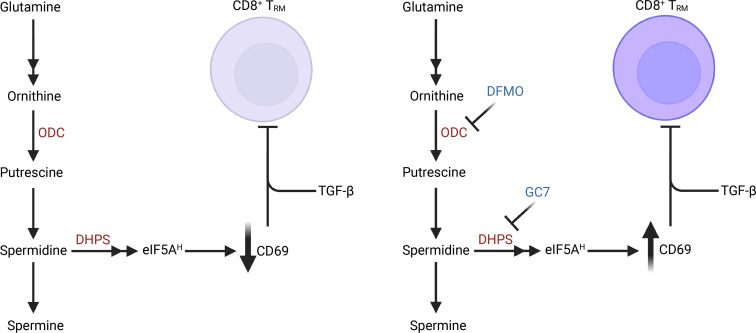

## Introduction

Metabolic reprogramming accompanies T cell activation, where naive CD8^+^ T cells switch from relying primarily on oxidative phosphorylation to glycolysis and glutaminolysis to meet the increased anabolic and energetic demands of proliferating and differentiating effector T cells (1). Glutamine is essential for T cell proliferation, cytokine production, and signaling (2), and T cell activation results in increases in glutamine uptake (2). Conversely, blocking glutamine catabolism has recently been shown to drive T cells toward highly activated, memory-like cells that have superior antitumor function (3, 4).

MYC is a master transcriptional regulator of T cell function and reprograms T cell metabolism following antigen activation (5). This metabolic reprogramming includes increases in glutamine uptake via upregulation of glutamine transporters, glutaminolysis, and anaplerotic flux of glutamate into the TCA cycle (2, 5, 6). Furthermore, this also includes the conversion of glutamate into ornithine, the substrate for biosynthesis of the polyamines, putrescine, spermidine, and spermine (5). Notably, the Myc/glutamine/polyamine axis is essential for activation-induced T cell proliferation (5, 7).

Ornithine decarboxylase (ODC) catalyzes the first and rate-limiting step of polyamine biosynthesis by converting ornithine to putrescine. Subsequently, spermidine synthase (SRM) converts putrescine to spermidine, and spermine synthase (SMS) then converts spermidine to spermine. Polyamines are polycationic amines that are known to play key roles in T cell proliferation and function (8–10). MYC induces the transcription of *ODC*, *SMS*, and *SRM* to promote polyamine biosynthesis (5, 11, 12). In eukaryotes, the aminobutyl group of spermidine is used to generate hypusine by 2 enzymes, deoxyhypusine synthase (DHPS) and deoxyhypusine hydroxylase (DOHH), which covalently link hypusine to a single conserved lysine residue in eukaryotic translation initiation factor 5A (eIF5A) and its ortholog eIF5A2 (13, 14). Importantly, this posttranslational modification facilitates peptide bond formation during translation elongation as well as termination of translation by promoting nascent peptide release (15).

Coinhibition of ODC enzyme activity and polyamine transport (so-called polyamine blocking therapy) has been shown to augment CD8^+^ T cell antitumor immunity (16). However, how the polyamine/hypsuine axis regulates CD8^+^ T cell fate is unclear. Here, we report that this axis tightly regulates CD69 expression, a cell-surface molecule that plays a key role in homing of tissue-resident memory T (Trm) cells to the BM niche (17, 18). Importantly, Trm cells play key roles in immunity (19, 20), and our studies reveal that the glutamine/polyamine/hypusine axis restricts the development of Trm cells and that it can be inhibited to augment the production and function of Trm cells.

## Results

### TCR-specific activation augments production of polyamines in CD8^+^ T cells.

**Figure 1:**
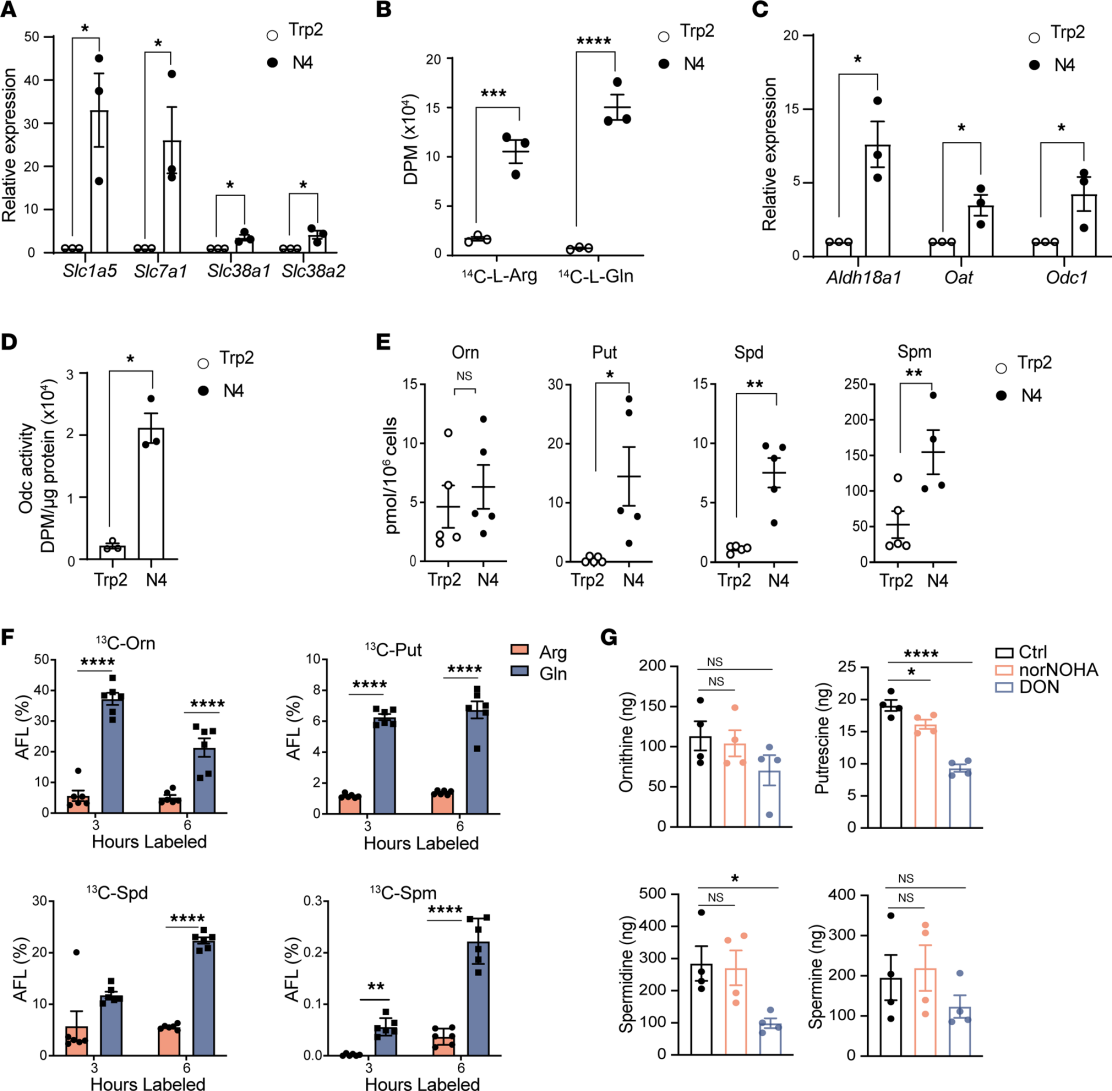
Polyamine metabolism is upregulated following mouse CD8^+^ T cell activation, and polyamines are primarily derived from glutamine. Transgenic OT-I T cells were stimulated with cognate antigen peptide SIINFEKL (N4) or an irrelevant peptide (Trp2) for 24 hours and the following were assessed. (**A**) Fold change in levels of the indicated mRNAs (determined by qPCR) normalized to *B2m* mRNA (*n* = 3). (**B**) Uptake of ^14^C-L-Arg and ^14^C-L-Gln as disintegrations per minute (DPM) (*n* = 3). (**C**) Fold change in levels of the indicated mRNAs (determined by qPCR) normalized to *B2m* mRNA (*n* = 3). (**D**) ODC enzyme activity (*n* = 3). (**E**) Levels of intracellular ornithine (Orn), putrescine (Put), spermidine (Spd), and spermine (Spm), as determined by liquid chromatography–mass spectrometry (*n* = 5). (**F**) Apparent fractional labeling (AFL%) of ornithine, putrescine, spermidine, and spermine after 3- or 6-hour labeling of arginine or glutamine (*n* = 6). (**G**) Levels of ornithine and the polyamines 300 μM nor-NOHA or 3 μM of DON (*n* = 4) after 24 hours. Data in **A**–**C** and **F** were analyzed using 2-way ANOVA and Dunnett’s multiple-comparison test, data in **D** and **E** were analyzed by unpaired *t* test, and data in **G** were analyzed by 1-way ANOVA and Dunnett’s multiple-comparison test. In all panels, each dot indicates a biologically independent sample, and data are shown as mean ± SEM. *P < 0.05; **P < 0.01; ***P < 0.001; ****P < 0.0001.

To assess polyamine metabolism in activated T cells, we used splenic CD8^+^ T cells from the OT-I transgenic mouse that expresses a T cell receptor (TCR) transgene specific for the ovalbumin (OVA) peptide SIINFEKL (OVA amino acid residues 257–264) (21). Using SIINFEKL and an irrelevant peptide derived from tyrosinase-related protein 2 (Trp2 amino acid residues 180–188), we confirmed (22, 23) that antigen-specific activation induces the expression of several amino acid transporters (Figure 1A), including the glutamine transporter *Slc1a5*, the arginine cation transporter *Slc7a1*, and 2 glutamine transporters relevant to T cell biology, *Slc38a1* and *Slc38a2* (24, 25). Arginine and glutamine can potentially serve as carbon sources in the biosynthesis of polyamines, and uptake assays confirmed antigen-specific induction of radiolabeled ^14^C-L-arginine and ^14^C-L-glutamine uptake in OT-I T cells (Figure 1B).

Following the conversion of glutamine to glutamate (which is directed by glutaminase [GLS1]), glutamate is reduced to Δ^1^-pyrroline-5-carboxylate (P5C) by the aldehyde dehydrogenase ALDH18A1. P5C is then converted to ornithine by ornithine aminotransferase (OAT); then, ornithine is converted to putrescine by ODC. Notably, quantitative PCR (qPCR) expression analyses revealed that antigen activation of OT-I cells was associated with a significant induction of all 3 enzymes (Figure 1C). Furthermore, ODC enzyme activity was also significantly increased following antigen activation of CD8^+^ OT-I T cells (Figure 1D). Finally, although total intracellular ornithine levels were not increased after activation, total putrescine, spermidine, and spermine levels were significantly elevated (Figure 1E). Thus, polyamine production is coordinately induced following antigen-specific TCR activation of CD8^+^ T cells.

### Polyamines are primarily derived from glutamine in antigen-activated CD8^+^ T cells.

Given that both arginine and glutamine uptake are significantly induced following TCR stimulation (Figure 1B), activated CD8^+^ T cells were labeled with ^13^C-arginine or ^13^C-glutamine to fully label the intracellular arginine and glutamine pools (Supplemental Figure 1, A and B; supplemental material available online with this article; https://doi.org/10.1172/jci.insight.169308DS1). Activated OT-I T cells labeled with ^13^C-glutamine had significantly higher incorporation of labeled carbons into ornithine, putrescine, spermidine, and spermine versus OT-I T cells labeled with ^13^C-arginine (Figure 1F). In accord with this finding, total intracellular levels of glutamine were significantly decreased following activation of OT-I T cells when compared with arginine levels (Supplemental Figure 1C); thus, the carbons of polyamines are primarily derived from glutamine.

To confirm that polyamines are principally derived from glutamine, activated CD8^+^ T cells were cultured with the arginase inhibitor N^ω^-hydroxy-nor-L-arginine (nor-NOHA) or with the GLS1 inhibitor 6-diazo-5-oxo-L-norleucine (DON). As expected, intracellular putrescine and spermidine were significantly decreased in SIINFEKL peptide–activated OT-I cells following treatment with DON but were not affected by nor-NOHA treatment (Figure 1G).

To assess if arginine contributed to polyamine pools following glutamine deprivation or inhibition of GLS1, we measured the uptake of arginine and ornithine — and the intracellular levels of arginine, ornithine, and the polyamines — when CD8^+^ T cells were activated in glutamine-deficient media or in the presence of DON. Putrescine and spermidine levels decreased following glutamine deprivation or DON treatment, yet arginine pools remained unchanged (Supplemental Figure 1D). Finally, CD8^+^ T cells don’t compensate for glutamine limitation or inhibition of GLS1 by increasing the uptake of ^14^C-L-ornithine or ^14^C-L-arginine (Supplemental Figure 1E). Thus, polyamine biosynthesis is primarily driven by glutamine catabolism in antigen-activated CD8^+^ T cells.

### The glutamine/polyamine/hypusine axis controls CD69 expression.

**Figure 2:**
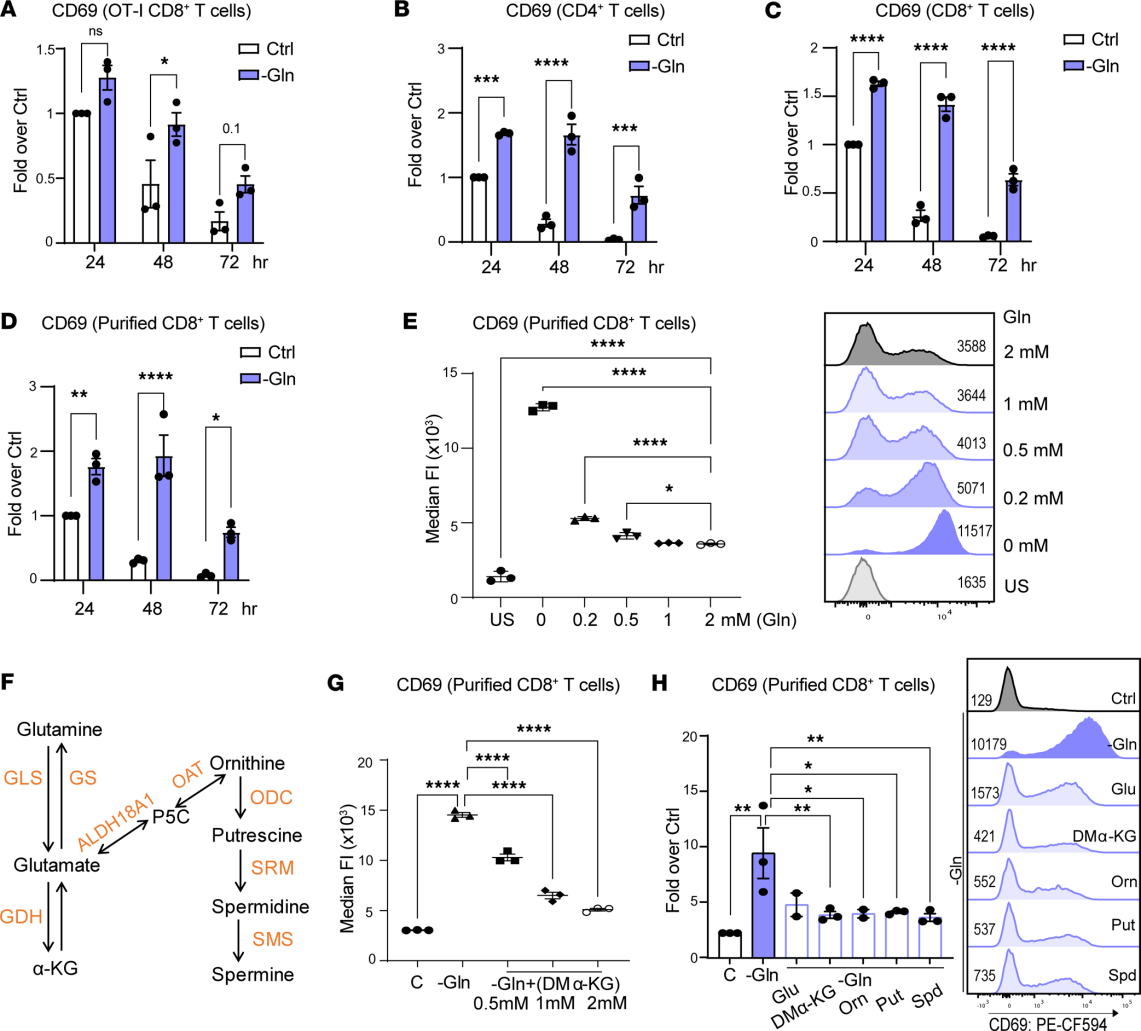
The glutamine-polyamine pathway controls CD69 expression in activated mouse CD8^+^ T cells. (**A**) CD69 MFI in CD8^+^ OT-I T cells at 24, 48, and 72 hours after activation in replete versus glutamine-deficient media (*n* = 3). (**B** and **C**) CD69 MFI in activated polyclonal CD4^+^ T cells (**B**) and CD8^+^ T cells, respectively (*n* = 3) (**C**). (**D**) CD69 MFI in purified CD8^+^ T cells at 24, 48, and 72 hours after activation (*n* = 3), in replete versus glutamine-deficient media. Ctrl, control complete RPMI media with 2 mM glutamine; –Gln, Glutamine-deficient media. (**E**) CD69 MFI in CD8^+^ T cells cultured in media having the indicated concentrations of glutamine (0, 0.2, 0.5, 1, and 2 mM) 72 hours after activation. US, unstimulated. (**F**) Schematic of the metabolic network connecting glutamine to the polyamine pathway. (**G**) CD69 MFI in CD8^+^ T cells cultured in replete media (denoted as C) or in glutamine-deficient media treated with the indicated concentrations of DM-KG (0.5, 1, and 2 mM) 72 hours after activation. (**H**) CD69 MFI in CD8^+^ T cells cultured in replete media (denoted as C) or in glutamine-deficient media ± 2 mM DM-KG, 1 mM ornithine, 500 μM putrescine (Put), or 100 μM spermidine (Spd). Data in **A**–**D** were analyzed using 2-way ANOVA with Dunnett’s multiple-comparison test. Data in **E**, **G**, and **H** were analyzed by 1-way ANOVA and Dunnett’s multiple-comparison test. In **A**–**D** and **H**, each dot represents a biological replicate, and data are shown as mean ± SEM. In **E** and **G**, each dot represents a technical replicate, and data are shown as mean ± SD. *P < 0.05; **P < 0.01; ***P < 0.001; ****P < 0.0001.

To assess the role of the glutamine/polyamine axis in controlling the phenotype and function of CD8^+^ T cells, we initially evaluated the effects of glutamine depletion on the expression of CD69, an important marker of memory T cells that is expressed early following T cell activation and is then downregulated (17, 18, 26). OT-I cells were activated using SIINFEKL in glutamine replete medium (i.e., RPMI containing 2 mM glutamine), or in glutamine-deficient medium, and levels of CD69 were assessed by flow cytometry. Interestingly, CD69 expression was augmented and sustained under glutamine-deficient conditions following antigen activation of OT-I T cells (Figure 2A) as well as following activation of purified splenic naive CD4^+^ and CD8^+^ T cells from C57BL/6J mice with anti-CD3 and anti-CD28 stimulation (Figure 2, B and C). Glutamine depletion provoked significant increases in cell-surface expression of CD69 in both CD4^+^ and CD8^+^ T cells, as reflected by the increase in median fluorescence intensity (MFI), even at early time points (24 and 48 hours) following CD3/CD28 engagement (Supplemental Figure 2A).

To assess whether sustained CD69 expression in CD8^+^ T cells was independent of CD4^+^ T cell help, CD8^+^ T cells were purified and then activated in glutamine-deficient media. Again, glutamine depletion provoked significantly increased CD69 levels compared with glutamine replete conditions (Figure 2D and Supplemental Figure 2B), and conversely, activation of CD8^+^ T cells in increasing concentrations of glutamine resulted in significant, dose-dependent reductions in CD69 expression (Figure 2E).

Glutamine can feed either into the tricarboxylic acid (TCA) cycle as α-ketoglutarate (αKG) or into the polyamine pathway via P5C (Figure 2F). To assess if glutamine-derived metabolites could reverse the effects of glutamine depletion on CD69 expression, we initially tested the effects of dimethyl-αKG (DM-KG), a cell-permeable form of αKG. DM-KG treatment resulted in significant, dose-dependent reductions in CD69 expression in glutamine-deprived activated CD8^+^ T cells (Figure 2G). However, since conversion of glutamate to KG is reversible (27) and could fuel the polyamine pathway, we also assessed the effects of adding glutamate or ornithine. Notably, supplementation with either of these metabolites significantly reduced cell surface levels of CD69 in glutamine-deprived activated CD8^+^ T cells (Figure 2H). Importantly, adding back the polyamines putrescine, or spermidine also completely reversed the effects of glutamine deprivation on CD69 expression in activated in CD8^+^ T cells (Figure 2H).

**Figure 3:**
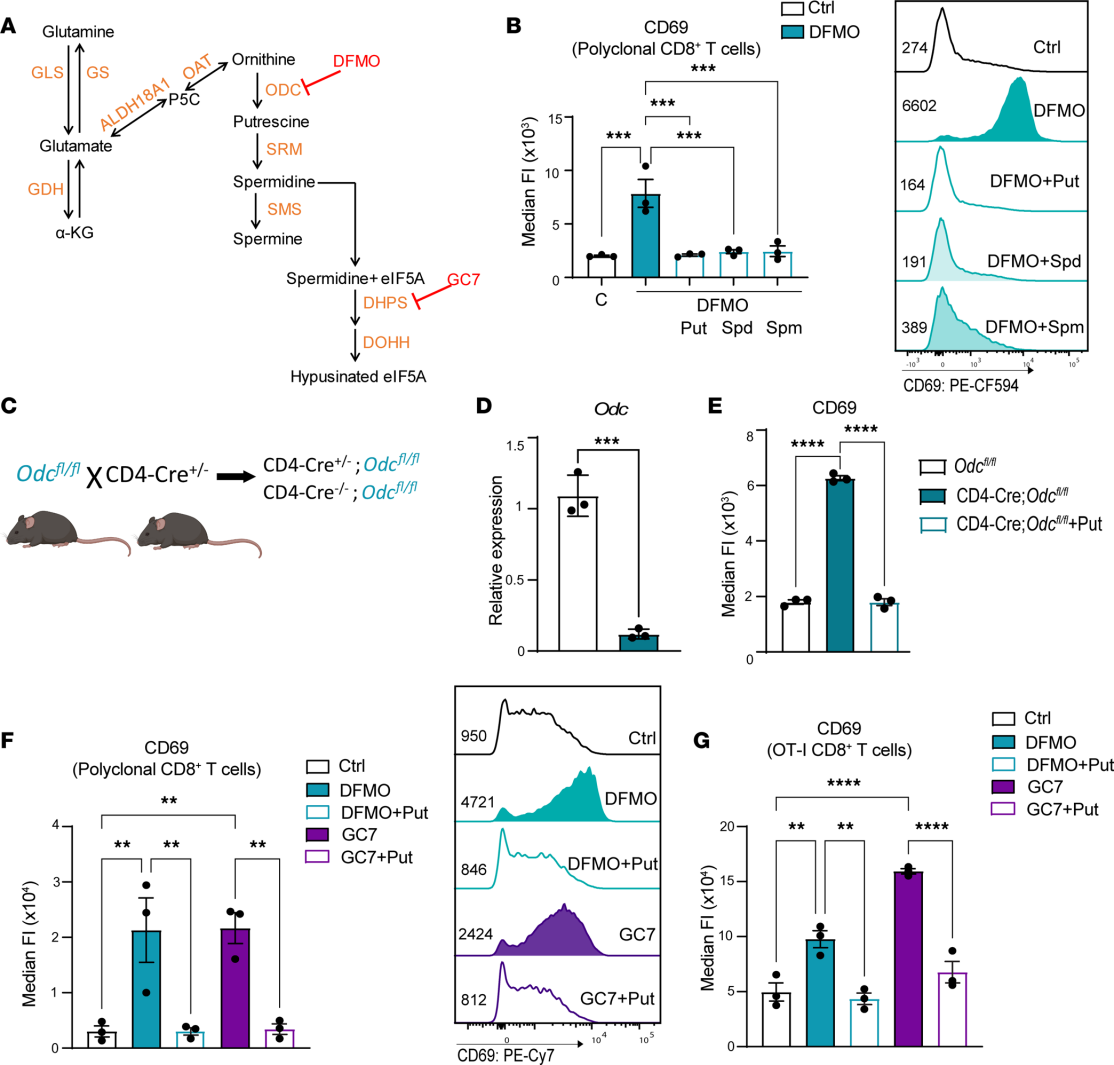
The polyamine/hypusine axis suppresses CD69 expression in activated mouse CD8^+^ T cells. (**A**) Schematic of the polyamine-hypusine pathway and selected pharmacologic inhibitors. (**B**) CD69 MFI in polyclonal CD8^+^ T cells activated in replete RPMI-1640 medium (with 2 mM glutamine) without (Ctrl) or with added 5 mM DFMO ± 500 μM putrescine (Put), 100 μM spermidine (Spd), or 100 μM spermine (Spm) 72 hours after activation (*n* = 3). (**C**) Breeding scheme for the generation of the CD4-Cre^+/–^;*Odc^fl/fl^* mice. (**D**) Fold change in levels of *Odc* mRNA (determined by qPCR) normalized to *B2m* mRNA in CD8^+^ T cells from CD4-Cre^+/–^;*Odc^fl/fl^* mice (*n* = 3). (**E**) CD69 MFI in CD8^+^ CD4-Cre^+/–^;*Odc^fl/fl^* T cells ± 500 μM Put (*n* = 3). (**F** and **G**) CD69 MFI in purified polyclonal CD8^+^ T cells (**F**), or CD8^+^ OT-I T cells (**G**), activated under control (Ctrl) conditions or with added 5 mM DFMO ± Put or 10 μM GC7 ± Put at 72 hours after activation (*n* = 3). Data in **B** and **E**–**G** were analyzed by 1-way ANOVA with Tukey’s post hoc test, and data in **D** were analyzed using unpaired *t* test. Each dot represents a biological replicate, and all data are shown as mean ± SEM. **P < 0.01; ***P < 0.001; ****P < 0.0001.

To confirm that polyamines control CD69 expression, we tested the effects of inhibiting ODC using difluoromethylornithine (DFMO), an irreversible suicide inhibitor of ODC (28) (Figure 3A). Similar to glutamine deprivation, DFMO treatment resulted in increased and sustained CD69 expression in activated CD8^+^ T cells, and the stimulatory effects of DFMO were abolished by cotreatment with polyamines (Figure 3B). Intracellular polyamine pools are also controlled by active transport (29), and polyamine uptake is induced following inhibition of ODC by DFMO (30). Trimer44NMe is an effective polyamine transport inhibitor (PTI) (31, 32), and we therefore tested whether further depleting polyamines by preventing the uptake of extracellular polyamines would increase cell-surface levels of CD69 in activated CD8^+^ T cells. Although PTI treatment alone had no effect on CD69 levels, PTI cotreatment with DFMO further increased CD69 levels in activated CD8^+^ T cells (Supplemental Figure 3A).

To confirm these findings, we generated mice that lack *Odc* in CD8^+^ T cells. CD4-Cre mice, which first express Cre recombinase in CD4^+^CD8^+^ double-positive thymocytes during T cell development (33), were crossed to the *Odc^fl/fl^*–conditional KO mouse model (34) to generate CD4-Cre*;Odc^fl/fl^* mice (Figure 3C). Characterization of CD4-Cre*;Odc^fl/fl^* mice revealed that they have normal numbers of splenic CD4^+^ and CD8^+^ T cells (not shown) and that, as expected, splenic CD4-Cre*;Odc^fl/fl^* CD8^+^ T cells expressed nearly undetectable levels of *Odc* mRNA (Figure 3D). Notably, activation of splenic CD8^+^ T cells from CD4-Cre*;Odc^fl/fl^* mice using CD3/CD28 dynabeads resulted in sustained expression of CD69 that was reversed by putrescine addback, confirming that polyamines controlled CD69 expression (Figure 3E).

Spermidine is also a substrate for a unique posttranslational modification coined hypusination, which occurs on a single lysine residue (lysine-50) in the translation factor eIF5A to augment translation elongation, and this modification is directed by 2 essential enzymes, DHPS and DOHH (13) (Figure 3A). To test the role of eIF5A hypusination in controlling CD69 levels, activated CD8^+^ T cells were treated with a reversible inhibitor of DHPS coined GC7 (N1-guanyl-1,7-diaminoheptane), which is a spermidine analogue (Figure 3A) (35). Similar to the effects of DFMO, GC7 treatment increased and sustained cell-surface CD69 expression in anti-CD3/CD28–activated CD8^+^ T cells (Figure 3F). Furthermore, these effects were inhibited by cotreatment with putrescine, which is converted to spermidine to competitively block GC7 activity (Figure 3F). Similar findings were manifest in antigen-activated CD8^+^ OT-I T cells (Figure 3G). Thus, the glutamine/polyamine/hypusine axis controls CD69 expression in activated CD8^+^ T cells.

Methionine also contributes to spermidine and spermine biosynthesis in activated CD8^+^ T cells at 2 levels. First, the expression of methionine transporter *Slc7a5* is increased following antigenic stimulation of OT-I T cells (Supplemental Figure 3C). Second, methionine contributes aminopropyl groups to synthesis of spermidine and spermine following: (a) conversion to S-adenosylmethionine (SAM) by methionine adenosyltransferase (MAT2); (b) decarboxylation of SAM to decarboxylated SAM (dcSAM) by SAM decarboxylase 1 (AMD1); and (c) use of the aminopropyl groups of dcSAM by SRM and SMS to generate spermidine and spermine, respectively (Supplemental Figure 3B). Notably, inhibition of AMD1 by treatment with the selective inhibitor SAM486A (36) significantly reduced methionine uptake in activated CD8^+^ T cells (Supplemental Figure 3D), as well as levels of spermidine and spermine, but resulted in significant increases in putrescine levels (Supplemental Figure 3E). Importantly, AMD1 inhibition resulted in sustained levels of CD69 in activated CD8^+^ T cells that were rescued by adding back spermidine but not by adding back putrescine (Supplemental Figure 3F). Thus, spermidine-derived hypusination downregulates CD69 expression in activated CD8^+^ T cells.

To gain insight into how the polyamine/hypusine axis regulates CD69, a parallel time course analyses of CD69 cell surface protein and *CD69* mRNA levels was performed in activated CD8^+^ T cells and in these cells treated with DFMO or GC7. Increased levels of cell-surface CD69 in DFMO- and GC7-treated activated CD8^+^ T cells was first evident at 24 hours after activation, and increased levels were sustained at 96 hours after activation (Supplemental Figure 3G). In contrast, there was little effect of DFMO or GC7 treatment on levels of *CD69* mRNA (Supplemental Figure 3H), suggesting that control of CD69 occurs largely at a posttranscriptional level.

### The glutamine/polyamine/hypusine axis controls CD69 expression in human CD8^+^ T cells, sarcoma TIL, and CAR-T cells.

**Figure 4:**
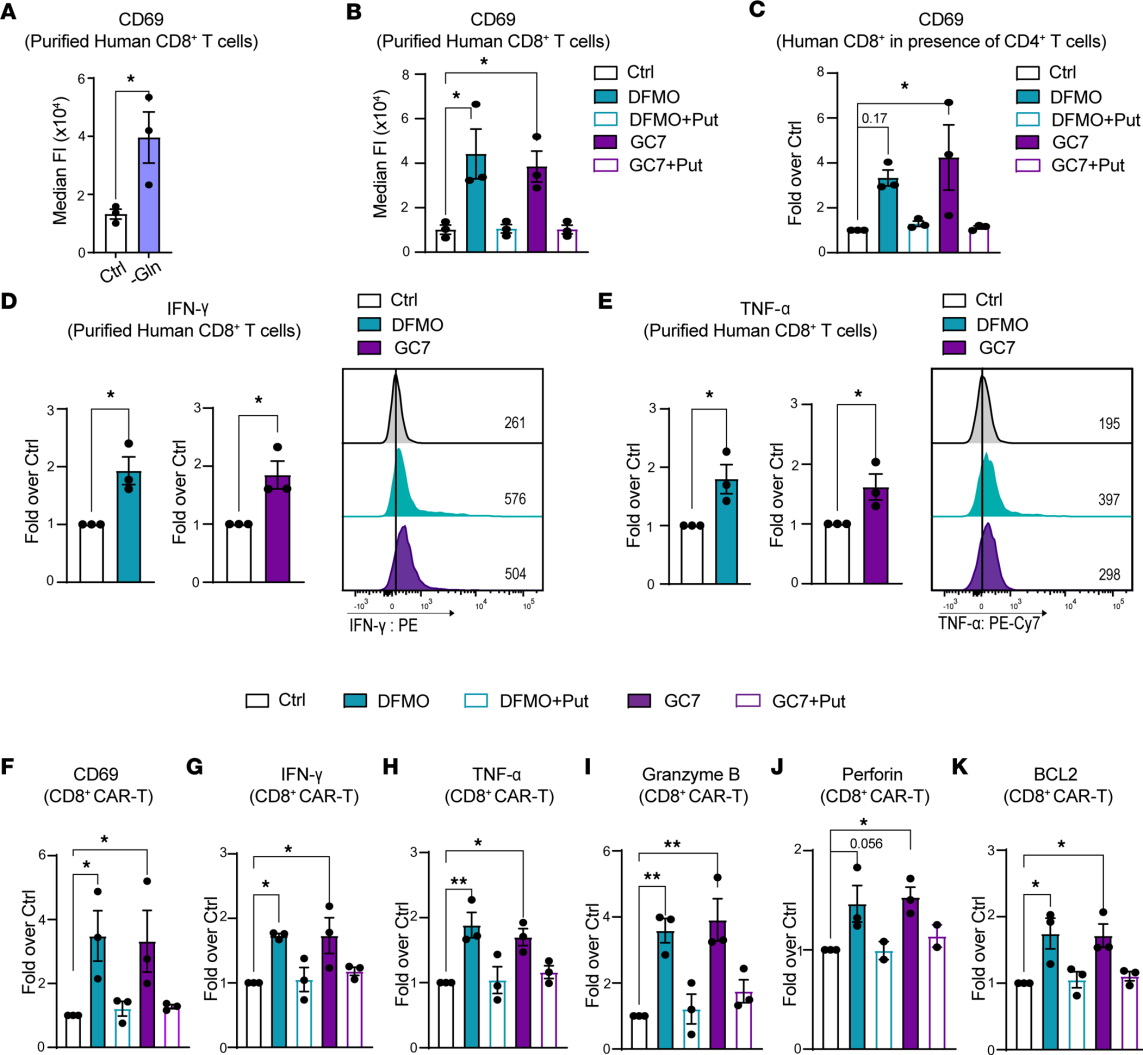
Glutamine/polyamine/hypusine axis controls CD69 expression in activated human CD8^+^ T cells. (**A**) CD8^+^ human T cells isolated from PBMC were activated with anti-CD3/CD28 and cultured replete media (Ctrl) or glutamine-deficient media (–Gln) and were analyzed for CD69 expression after 7 days (*n* = 3). (**B** and **C**) Purified CD8^+^ T cells (**B**) or CD8^+^ T cells from pan T cells from PBMC (**C**) were activated under control (Ctrl) conditions or were treated with 5 mM DFMO ± Put (500 μM) or with 10 μM GC7 ± Put (500 μM), and CD69 was expression analyzed after 7 days (*n* = 3). (**D** and **E**) MFI of IFN-γ (**D**) and TNF-α (**E**) in purified human CD8^+^ T cells from PBMC that were activated under control conditions (Ctrl) or that were treated with 5 mM DFMO or 10 μM GC7 for 7 days (*n* = 3). (**F**–**K**) MFI of CD69 (**F**), IFN-γ (**G**), TNF-α (**H**), granzyme B (**I**), perforin (**J**), and BCL2 (**K**) in human CD19–targeting CD8^+^ CAR-T cells cultured in RPMI media supplemented with IL-2 and treated with 5 mM DFMO or 10 μM GC7 ± 500 μM putrescine for 7 days (*n* = 3). Data in **A**, **D**, and **E** were analyzed using unpaired *t* test. Data in **B**, **C**, and **F**–**K** were analyzed using 1-way ANOVA and Dunnett’s multiple-comparison test. Each dot represents a biological replicate, and all data are shown as mean ± SEM. *P < 0.05; **P < 0.01.

Given that the glutamine/polyamine/hypusine axis regulates CD69 in mouse CD8^+^ T cells, we assessed the effects of this pathway in human CD8^+^ T cells. Activation of human CD8^+^ T cells (from normal PBMC) in glutamine-deficient media sustained CD69 levels 7 days after activation (Figure 4A). Furthermore, inhibition of ODC or DHPS using DFMO or GC7, respectively, resulted in sustained CD69 levels in activated human CD8^+^ T cells at day 7 after activation, using either purified CD8^+^ T cells or CD8^+^ T cells cultured in the presence of CD4^+^ T cells (Figure 4, B and C). Similar to findings in primary mouse CD8^+^ T cells, adding back putrescine reduced CD69 levels to those found under control conditions in DFMO- or GC7-treated activated human CD8^+^ T cells (Figure 4, B and C). In addition, DFMO and GC7 treatment sustained CD69 expression in CD8^+^ T cells from human sarcoma tumor infiltrating lymphocytes (TIL) following activation with anti-CD3/CD28 and IL-2 (Supplemental Figure 4, A and F). Inhibition of the polyamine/hypusine axis also increased the polyfunctionality of primary CD8^+^ T cells and of CD8^+^ TIL, where activation in the presence of DFMO or GC7 increased the production of IFN-γ and TNF-α (Figure 4, D and E, and Supplemental Figure 4, B–D, G, and H). Furthermore, upon gating for CD69^+^ TIL, there was an increase in IFN-γ and TNF-α production in DFMO-treated TIL (Supplemental Figure 4E). Finally, DFMO or GC7 treatment of human CD19–targeting CD8^+^ CAR-T cells also led to significant increases in CD69, IFN-γ, TNF-α, and BCL2 levels and augmented their production of the cytotoxic effector proteins perforin and granzyme B (Figure 4, F–K). Thus, the polyamine-hypusine pathway also controlled CD69 cell surface expression in human CD8^+^ T cells, and disabling this circuit augmented their function, underscoring the potential of targeting the polyamine/hypusine axis to improve adoptive T cell therapies.

### Modulating the polyamine-hypusine circuit can promote differentiation of CD8^+^ Trm cells.

**Figure 5:**
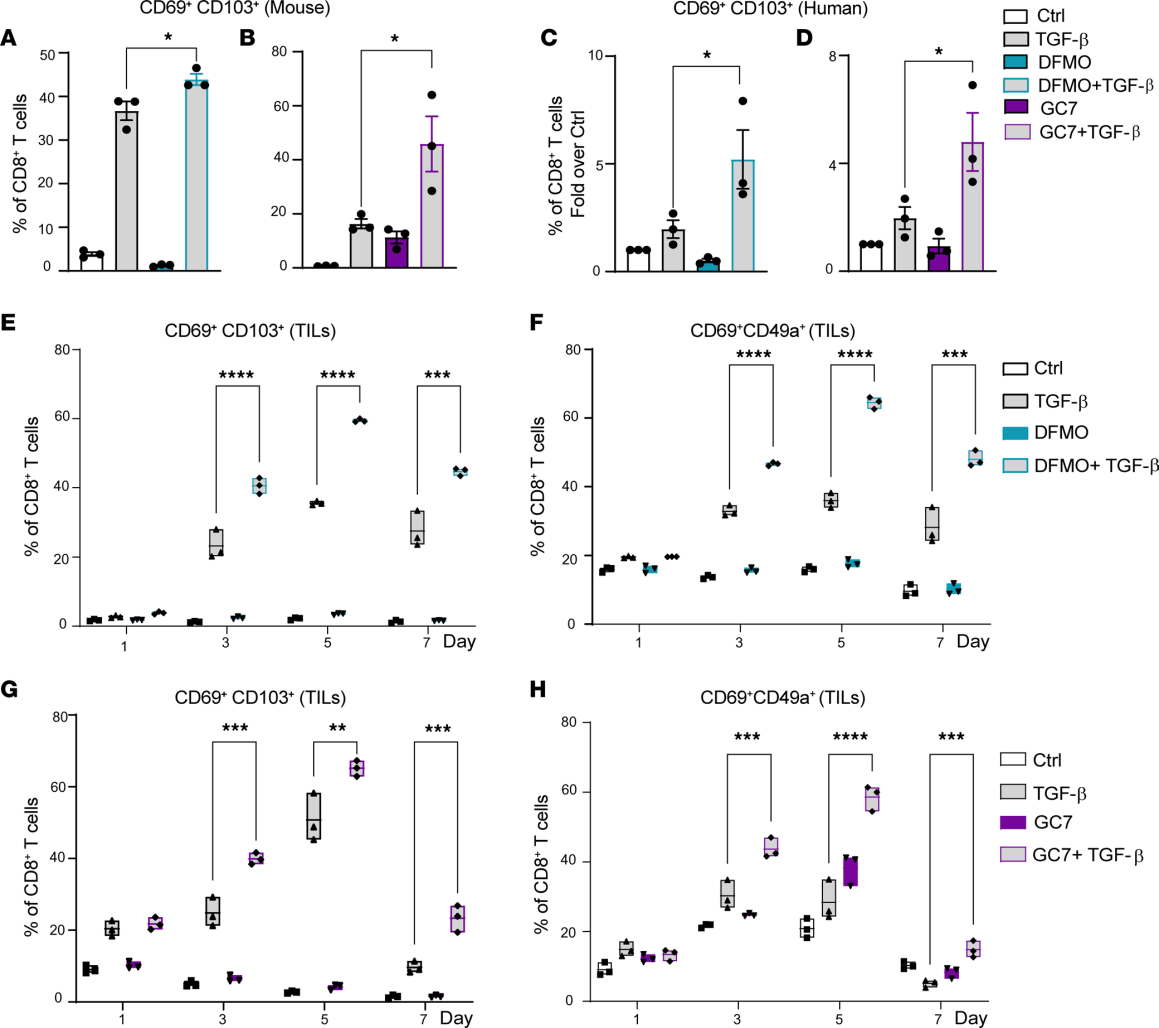
Inhibition of the polyamine/hypusine axis augments the generation of Trm cells ex vivo. (**A** and **C**) The percentage of CD69^+^CD103^+^ in CD8^+^ T cells that were untreated (Ctrl) or were treated with 10 ng/mL TGF-β and/or 5 mM DFMO at day 3 (mouse) (**A**) or day 7 (human) (**C**) following activation with anti-CD3/CD28 (*n* = 3). (**B** and **D**) The percentage of CD69^+^CD103^+^CD8^+^ T cells from control cultures (Ctrl) or cells treated with 10 ng/mL TGF-β and/or 10 μM GC7 at day 3 (mouse) (**B**) or day 7 (human) (**D**) after activation (*n* = 3). (**E** and **F**) Percentages of CD69^+^CD103^+^ (**E**) and CD69^+^CD49a^+^ (**F**) CD8^+^ Trm cells in human sarcoma post-REP TIL activated in Ctrl media 10 ng/mL TGF-β or treated with 5 mM DFMO 10 ng/mL TGF-β for 7 days. (**G** and **H**) Percentages of CD69^+^CD103^+^ (**G**) and CD69^+^CD49a^+^ (**H**) CD8^+^ Trm cells from human sarcoma post-REP TIL activated in Ctrl media 10 ng/mL TGF-β or treated with 10 μM GC7 10 ng/mL TGF-β for 7 days. Each dot in **A**–**D** represents a biological replicate, and data were analyzed using 1-way ANOVA with Tukey’s post hoc test and are shown as mean ± SEM. Data in **E**–**H** are representative of 3 independent experiments and were analyzed using multiple *t* tests with Holm-Šídák test and are shown as mean ± SD. *P < 0.05; **P < 0.01; ***P < 0.001; ****P < 0.0001.

CD69 plays a crucial role in the development of Trm cells by preventing T cell egress from tissues (37, 38), and it works in concert with other niche cues such as TGF-β to drive the differentiation of CD69^+^CD103^+^ Trm cells (4). To test if we could augment the generation of Trm cells by blocking the polyamine/hypusine axis, we activated mouse CD8^+^ T cells with TGF-β only or in combination with DFMO or GC7. Notably, combined treatment of activated mouse splenic CD8^+^ T cells, or of activated human CD8^+^ T cells, with DFMO or GC7 with TGF-β led to an enrichment of CD69^+^CD103^+^ Trm cells (Figure 5, A–D). Interestingly, adding TGF-β to human sarcoma TIL treated with DFMO (Figure 5, E and F) or with GC7 (Figure 5, G and H) also enhanced the generation of CD69^+^CD103^+^ and CD69^+^CD49a^+^ Trm cells. Importantly, when gated on CD69^+^CD49a^+^ or CD69^+^CD103^+^ cells, DFMO cotreatment led to increased levels of IFN-γ and TNF-α in post–rapid expansion protocol (post-REP) human sarcoma TIL (Supplemental Figure 5, A and B). Thus, targeting the polyamine/hypusine axis promoted Trm CD8^+^ T cell differentiation.

### Inhibition of the polyamine/hypusine axis enhances the generation of BM CD8^+^ Trm cells.

**Figure 6:**
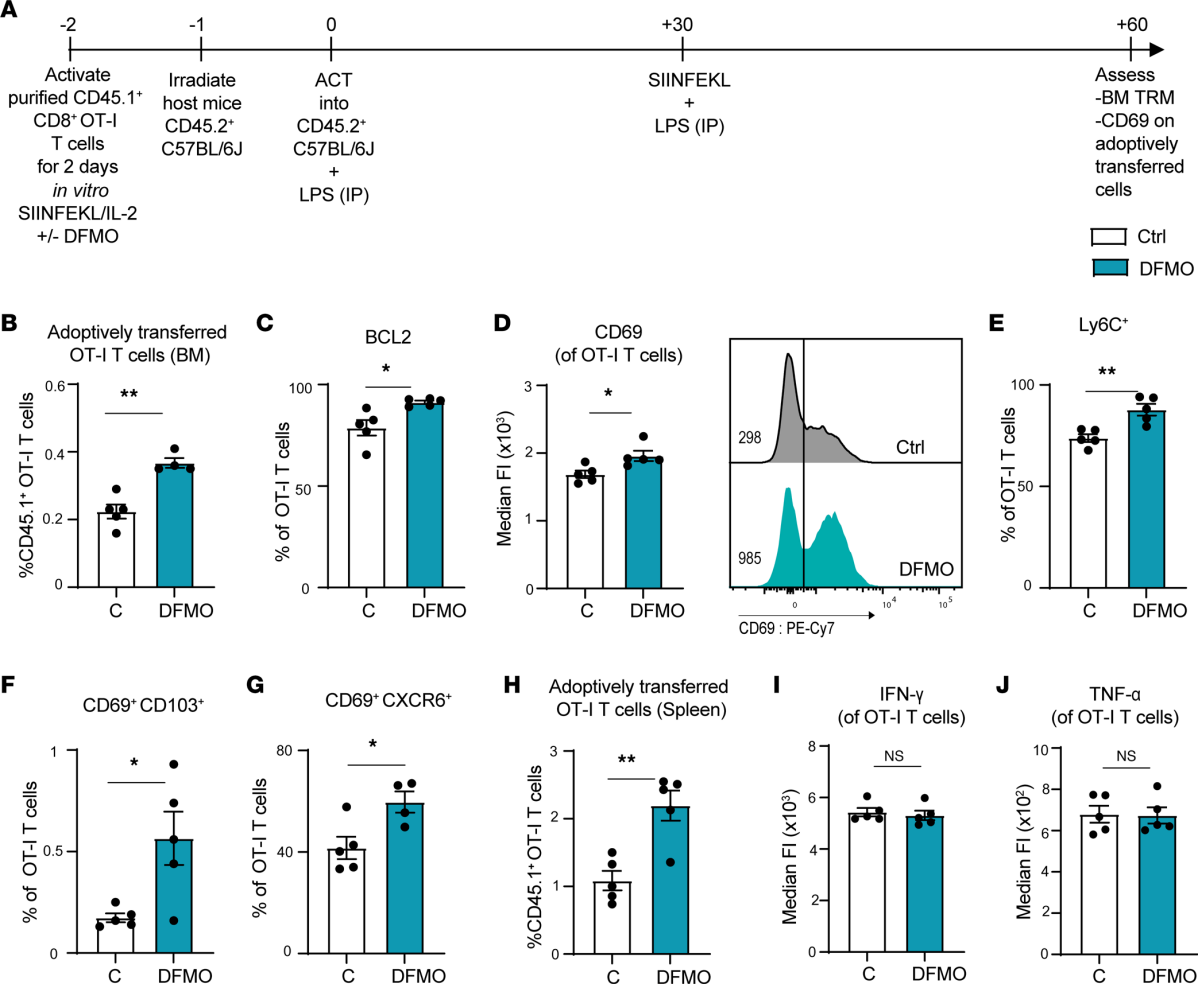
Inhibition of polyamine biosynthesis augments Trm cell generation in BM. (**A**) Schematic of the experimental design of the long-term BM CD8^+^ T cell ACT vaccination model. Two months following transplant of CD45.1^+^CD8^+^ OT-I T cells (stimulated with SIINFEKL and IL-2 ± 5 mM DFMO for 2 days prior to transplant) and 1 month following vaccination with SIINFEKL peptide + LPS, BM cells of CD45.2^+^ recipient mice were assessed for percentage of CD45.1^+^ OT-I. (**B** and **C**) CD8^+^ T cells and CD8^+^BCL2^+^ T cells are shown. (**D**) MFI of CD69 in CD45.1^+^ OT-I T cells. (**E**–**G**) Percentages of Ly6C^+^, CD69^+^CD103^+^, and CD69^+^CXCR6^+^ of CD45.1^+^CD8^+^ OT-I T cells in the BM of CD45.2^+^ recipient mice. (**H**) Percentage of CD45.1^+^CD8^+^ OT-I T cells in the spleens of CD45.2^+^ recipient mice. (**I** and **J**) MFI of IFN-γ and TNF-α in CD45.1^+^ CD8^+^ OT-I BM T cells. Unpaired *t* test was used. Each dot represents a biological replicate, and all data are shown as mean ± SEM (*n* = 5). *P < 0.05; **P < 0.01.

BM CD8^+^ Trm cells have a CD69^+^ phenotype, and CD69 plays a key role in the development of Trm cells in this niche (17). To test if ODC inhibition can enhance the development of Trm cells in vivo, we activated CD45.1^+^ OT-I T cells with SIINFEKL with and without DFMO in vitro for 48 hours, and we then adoptively transferred these cells into sublethally irradiated CD45.2^+^ C57BL/6J mice. Recipient mice were treated with 10 μg LPS on the day of adoptive cell transfer (ACT) and were then vaccinated with 100 μg SIINFEKL peptide and 10 μg LPS 1 month later. Two months after ACT, BM cells were then harvested and assessed for CD45.1^+^ OT-I counts and CD69 expression (Figure 6A). Interestingly, 2 months after adoptive transfer, DFMO-treated OT-I T cells appeared endowed with a survival advantage, as evidenced by a higher percentage of these cells in the BM (Figure 6B) and an increase in the expression of the prosurvival molecule BCL2 (Figure 6C). Furthermore, the DFMO-treated cohort expressed elevated levels of the memory markers CD69 and Ly6C (Figure 6, D and E), and these cells had higher numbers of BM Trm cells that had CD69^+^CD103^+^ and CD69^+^CXCR6^+^ phenotypes (Figure 6, F and G); DFMO treated OT-I T cells also displayed elevated numbers in the spleens of the recipient mice 2 months after ACT (Figure 6H). However, there were no significant differences in the expression of intracellular IFN-γ and TNF-α (Figure 6, I and J).

To test if inhibition of polyamine biosynthesis also augments CD69 expression in BM CD8^+^ T cells in a short-term vaccination model, CD45.1^+^ OT-I T cells were activated with SIINFEKL in vitro with or without DFMO for 48 hours and were then adoptively transferred into sublethally irradiated CD45.2^+^ C57BL/6J mice. Recipient mice were then treated with 10 μg LPS on the day of adoptive transfer, and OT-I cells were analyzed 7 days later (Supplemental Figure 6A). Again, OT-I cells from the DFMO-treated cohort expressed higher levels of CD69 in the BM and spleens of recipient mice (Supplemental Figure 6, B and C), without affecting the numbers of BM and splenic donor CD45.1^+^ cells at this early time following transplant (Supplemental Figure 6, D and E).

**Figure 7:**
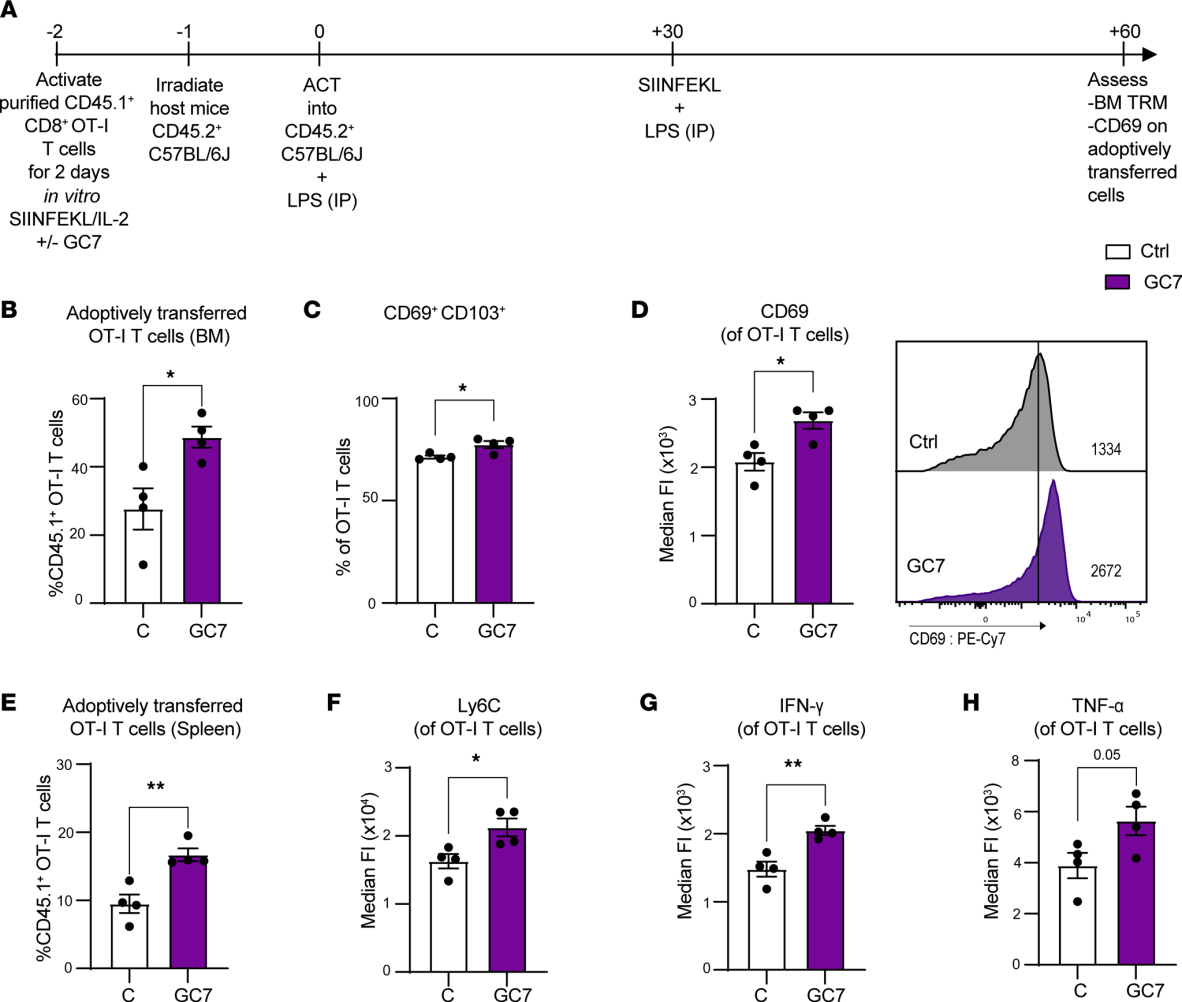
Inhibition of hypusination enhances Trm cell generation in BM in ACT vaccination model. (**A**) Schematic of the experimental design. (**B**–**E**) Two months following transplant of CD45.1^+^CD8^+^ OT-I T cells (stimulated with SIINFEKL and IL-2 ± 10 μM GC7 for 2 days prior to transplant) and 1 month following vaccination with SIINFEKL peptide + LPS, CD45.2^+^ recipient mice were assessed for percentage of BM CD45.1^+^CD8^+^ OT-I T cells (**B**); CD69^+^CD103^+^ OT-I T cells (**C**); MFI of CD69 in CD45.1^+^CD8^+^ OT-I T cells (**D**); and percentage of CD45.1^+^CD8^+^ OT-I T cells in the spleens of the CD45.2^+^ recipient mice (**E**). (**F**–**H**) MFI of Ly6C, IFN-γ, and TNF-α in CD45.1^+^CD8^+^ OT-I BM T cells. All data were analyzed by unpaired *t* test. Each dot represents a biological replicate, and all data are shown as mean ± SEM (*n* = 4). *P < 0.05; **P < 0.01.

To test if blocking hypusination also augments Trm formation in vivo, similar ACT and vaccination studies (Figure 7A) were performed with splenic CD45.1^+^ OT-I T cells treated with GC7 for 48 hours ex vivo before ACT. Notably, recipient mice receiving the GC7-treated cohort also had increased numbers of OT-I CD45.1^+^ cells in the BM and spleens of CD45.2^+^ recipient mice 2 months after ACT (Figure 7, B and E). In addition, the GC7-treated cohort also had increased numbers of CD69^+^CD103^+^ Trm cells in the BM (Figure 7C). OT-I cells from the GC7-treated cohort also displayed increased expression CD69 and Ly6C (Figure 7, D and F). Finally, transplanted GC7-treated OT-I cells also expressed increased levels of IFN-γ and TNF-α (Figure 7, G and H). Thus, inhibition of the polyamine-hypusine circuit augmented CD69 expression in CD8^+^ T cells in both short-and long-term in vivo settings and augmented the differentiation of BM Trm cells.

## Discussion

Activation-induced metabolic reprogramming of T cells is accompanied by increases in the uptake of essential nutrients, including glucose and amino acids. Of the latter, glutamine is critical for T cell function, as it provides — as underscored by the findings presented herein — precursors that enter into the TCA cycle via anaplerosis (39, 40) as well as those required for the biosynthesis of polyamines (5) that are also necessary for T cell proliferation (2). To support these needs, activated T cells increase glutamine uptake by upregulating the expression of glutamine transporters and enzymes involved in glutamine catabolism. For polyamine biosynthesis, this involves the conversion of glutamine to ornithine via a 2-step reaction that is catalyzed by ALDH18A1 and OAT. As shown here, antigen-specific activation of CD8^+^ T cells is associated with the induction of *Aldh18a1*, *Oat*, and *Odc* expression as well as with the induction of ODC enzyme activity. As a result, significant increases in levels of putrescine, spermidine, and spermine are observed, which isotope tracer analyses demonstrate are principally derived from glutamine rather than arginine.

Polyamines are polycationic alkyl amines that play many roles in cell physiology, including control of chromatin structure and stability, transcription, translation, and even ion channel function (41). ODC directs the rate-limiting step in polyamine biosynthesis by converting ornithine to putrescine, which is then converted to spermidine via SRM. Notably, the amino-butyl group of spermidine is a substrate for DHPS and DOHH enzymes that posttranslationally modify the translation elongation factor eIF5A via hypusination, which augments translation elongation and nascent peptide release during translation termination (42).

Recently, it has been shown that depletion of glutamine or inhibition of GLS1 augments the effector function and numbers of cytotoxic CD8^+^ T cells (4) and endows CD8^+^ T cells with a memory phenotype of enhanced activation, proliferation, and antitumor function (3). These findings are strikingly similar to those shown here, where blocking polyamine biosynthesis via treatment with the ODC inhibitor DFMO, or inhibiting eIF5A hypusination by treatment with the DHPS inhibitor GC7, phenocopies the effects of glutamine depletion on T cell activation. Specifically, glutamine depletion of antigen-activated CD8^+^ T cells, or their treatment with DFMO or GC7, leads to increased and sustained cell-surface expression of CD69, a regulator of Trm cells that provide protective immunity within key tissue niches, including BM (17, 18, 43). Furthermore, this phenotype is abolished by cotreatment with putrescine or spermidine, indicating that this response is dampened by the hypusine circuit that controls select translational programs of several biological processes (44, 45). Precisely how blocking eIF5A hypusination leads to increases and sustained expression of CD69 is the subject of current investigations, though the data do indicate that control is posttranscriptional.

Importantly, these findings also suggest that CD8^+^ T cell fate and function could be modulated by targeting the polyamine/hypusine axis and that one might exploit this circuit to drive the formation of Trm for therapeutic benefit. In support of this notion, blocking this axis increases the production of IFN-γ and TNF-α following activation of both mouse and human CD8^+^ T cells, and it augments TGF-β–induced differentiation of CD69^+^CD103^+^ Trm cells ex vivo. In an ACT model, ex vivo treatment of antigen-activated transgenic CD8^+^ OT-I T cells with either DFMO or GC7 for only 2 days was sufficient to increase numbers of Trm cells in both BM and spleen of transplanted recipient mice. Furthermore, these adoptively transferred cells have many of the hallmarks ascribed to Trm cells (46, 47), including increases in the proportion of BM CD69^+^CD103^+^, CD69^+^Ly6C^+^, and CD69^+^CXCR6^+^ CD8^+^ T cells that express BCL2 and, at least for GC7 transplanted cells, that produce increased levels of IFN-γ and TNF-α. Elevated production of IFN-γ and TNF-α is not sustained in DFMO-pretreated T cells, and this could reflect selective phenotype rescue in vivo. Regardless, the data establish that, in ACT models, blocking the hypusine axis before transplant was sufficient to enhance the generation of Trm-like (CD69^+^CD103^+^ and CD69^+^CXCR6^+^) T cells that stably expressed elevated levels of the Trm markers Ly6C and CD69 and that survived longer in the BM and spleen following transplant.

Underscoring the potential for exploiting the polyamine/hypusine axis for therapeutic benefit, ex vivo treatment of CAR-T cells or sarcoma post-REP TILs with GC7 or DFMO, a clinically approved agent for chemoprevention of prostate and colorectal cancer (48), augmented their production of IFN-γ and TNF-α, and either GC7 or DFMO treatment promotes TGF-β–induced differentiation of CD69^+^CD103^+^ and CD69^+^CD49a^+^ CD8^+^ Trm-like cells. Collectively, these findings support the premise that the polyamine-hypusine circuit can be exploited to augment the production and function of Trm cells to improve the efficacy of adoptive cellular therapies (e.g., CAR-T) that could be applied to ameliorate inflammation or treat autoimmune diseases and cancer.

## Methods

### Mice.

C57BL/6J (CD45.2^+^) and CD4-Cre mice (32) were purchased from The Jackson Laboratory (stock nos. 000664 and 022071, respectively), and the CD45.1^+^ OT-I mice were from the Pilon-Thomas laboratory (Moffitt Cancer Center). *Odc*^fl/fl^–conditional KO mice on a C57BL/6J background have been previously described (33). Mice were maintained in house. Biological replicates included 3–5 mice per cohort. All mice were 6–10 weeks old and were housed and bred in a specific pathogen–free animal facility.

### Cell culture, tissue processing, and mouse T cell isolation and activation studies.

For in vitro culture, spleens from 6- to 10-week-old mice were processed into single-cell suspension by dissociating with a 70 μm cell strainer and a syringe plunger in complete RPMI 1640 media (cRPMI; Thermo Fisher Scientific) with 10% FBS (GeminiBio, 900-108), 1% nonessential amino acids, 50 μM β-mercaptoethanol, 1% sodium pyruvate, and 1% penicillin/streptomycin (pen/strep) (all from Thermo Fisher Scientific). RBCs were then lysed using RBC lysis buffer (154 mM NH_4_Cl, 10 mM KHCO_3_, 0.1 mM EDTA in water; all from MilliporeSigma) for 1 minute. Afterward, 10 mL of complete media was added to stop the RBC lysis reaction. Splenocytes were counted by trypan blue dye (Thermo Fisher Scientific) exclusion using a Nexcelom automatic cell counter. Single-cell suspensions were placed in MACS buffer (PBS [Corning] plus 0.5% FBS, 0.5% BSA, and 2 mM EDTA [MilliporeSigma]).

Pan–CD3^+^ T cells or CD8^+^ T cells were isolated by immunomagnetic negative selection (Miltenyi Biotec; 130-095-130 [pan T cells], 130-104-075 [CD8^+^ T cells]) according to the manufacturer’s instructions. After labeling with biotin-antibody cocktail and anti-biotin microbeads sequentially, cells were placed in LS columns (Miltenyi Biotec, 130-042-401), and the negative selection fraction containing the T cells was collected for activation studies.

Mouse T cells were cultured in RPMI 1640 media supplemented with 10% FBS, 2 mM L-glutamine, 100 U/mL pen/strep, and 50 μM β-mercaptoethanol in a round-bottom 96-well plate at a seeding concentration of 2 × 10^5^ cells per well for polyclonal T cell activation. Activation of polyclonal T cells was performed using anti-CD3 and anti-CD28 Dynabeads (Dynabeads Mouse T-Activator CD3/CD28, Thermo Fisher Scientific, 11453D) at 1:1 bead/cell ratio and 10 ng/mL IL-2 (Peprotech). For CD8^+^ OT-I T cell activation, 1 × 10^6^ cells were plated per well in a 24-well plate with 0.1 nM OVA (residues 257–264) SIINFEKL peptide (Anaspec, AS-60193-1), or 0.1 nM Trp2 (residues 180–188) SVYDFFVWL peptide (Anaspec, AS-61058) and 10 ng/mL IL-2. Glutamine-deficient RPMI 1640 medium (Thermo Fisher Scientific, 21870076) was used for activation of T cells under no glutamine (–Gln) conditions. Cytokines were used at the following concentrations: 10 ng/mL for IL-2 (Peprotech) and 10 ng/mL for TGF-β (R&D Systems). Full manufacturer information for all products used in this study are provided in Supplemental Tables 2 and 3.

The following drug treatments were used: 5 mM DFMO, 10 μM GC7, and 10 μM of the AMD1 inhibitor SAM486A (sardomozide, HY-13746B), 500 μM putrescine hydrochloride (catalog P5780), 100 μM spermidine (catalog S2626), and 100 μM spermine (catalog S3256) (all from MilliporeSigma). In total, 200 μM aminoguanidine (MilliporeSigma, 2582301) was added alongside spermidine to block serum diamine oxidase. In total, 2 mM DM-KG (TCI America, 13192046) was also used for some addback studies. Glutamate (TCI America, 13515-99-6) and ornithine (MilliporeSigma, O2375) were used at 2 mM and 1 mM, respectively.

Drug treatments of 5 mM DFMO, 300 μM of the arginase inhibitor α–amino acid nor-NOHA, or 3 μM of the GLS1 inhibitor DON were added at the time of CD8^+^ T cell activation. Cells were then used for isotopic tracer experiments, polyamine quantification, qPCR, measurements of ODC enzyme activity, and amino acid uptake analyses. The Trimer44NMe PTI was dissolved in PBS to make a 10 mM stock solution, which was then filtered through a 0.2 μM filter for sterilization. Serial dilutions of Timer44NMe PTI were then made using PBS.

### In vivo BM ACT models.

Spleens, femurs, and tibias were isolated from recipient mice at day 7 (short-term BM ACT) and at day 60 (long-term BM ACT). Spleens were processed as described above, and bones were crushed using a mortar and pestle in complete media. RBC were lysed from single-cell suspensions of spleens and BM, which were then analyzed by flow cytometry.

### Human T cells from PBMCs.

Healthy human donor PBMCs were a gift from Javier Pinilla (Moffitt Cancer Center, Tampa, Florida, USA) and were either obtained from buffy coats (One Blood), isolated according to the manufacturer’s protocol, and frozen at –80°C in 90% FBS and 10% dimethylsulfoxide (DMSO) prior to use or were sourced from the Lifesouth Community Blood Centers (Location is Brooksville, Florida, USA). Human CD8^+^ T cells were isolated using negative immunomagnetic selection (Miltenyi Biotec, 130-096-495) and were cultured in RPMI 1640 media supplemented with 10% FBS and 100 U/mL pen/strep, cultured in 96-well plates at a seeding concentration of 2 × 10^5^ cells per well. Activation was performed using CD3/CD28 Dynabeads (Dynabeads Human T-Activator CD3/CD28, Thermo Fisher Scientific, 11131D) at 1:1 bead/cell ratio in the presence of 10 ng/mL IL-2 (PeproTech, 200-02).

### CAR construct and retroviral production.

Second-generation CAR constructs were synthesized and cloned into the pMXs (Cell Biolabs) retroviral vector from Genewiz (Azenta Life Sciences). CARs were constructed to contain a CD8a signal peptide, FMC63 scFv, a CD8a hinge and transmembrane domain, and CD28 and CD3z endodomains. To create stably transduced packaging cells, H29 packaging cells were transfected with the CAR construct using a calcium phosphate transfection kit (Invitrogen). After 5 days, retroviral supernatants were harvested, filtered (0.45 μm) to remove debris, and used to transduce RD114 packaging cells that have pseudotyped virus for human T cell infection. H29-derived retroviral supernatants were added to RD114 cultures with polybrene (Sigma-Adlrich) (0.08 μg/mL) to enhance transfection. RD114 retroviral supernatants were harvested, filtered (0.45 μm), and stored at –80°C.

### Primary T cell isolation and transduction with CARs.

Healthy donor T cells were isolated using a human T cell isolation kit (StemCell Technologies) per the manufacturer’s protocol. All T cells and CAR T cells were cultured in RPMI (Thermo Fisher Scientific) supplemented with 10% FBS, 100 IU IL-2/mL (R&D Systems), 100 U penicillin/mL (Thermo Fisher Scientific), and 100 μg/mL streptomycin (Thermo Fisher Scientific) referred to as cRPMI. Five experimental conditions were used to assess the effect of the polyamine/hypusine axis on CAR T cell differentiation and phenotype during manufacture: (a) cRPMI; (b) cRPMI supplemented with 5 mM DFMO; (c) cRPMI supplemented with 5 mM DFMO and 500 μM putrescine; (d) cRPMI supplemented with 10 μM GC7; and (e) cRPMI with 10 μM GC7 and 500 μM putrescine. Prior to transduction, enriched CD3 T cells were activated for 24 hours with anti-CD3/anti-CD28 dynabeads (Invitrogen) at a 1:1 T cell/bead in 1 of 5 of the media conditions above. After 24 hours, T cells were counted, resuspended in fresh media at a concentration of 0.5 × 10^6^ to 0.75 × 10^6^/mL, seeded into Retronectin-coated (Takara) plates with retroviral supernatant, and centrifuged for 1 hour at 2,000*g* and 32°C. T cells were infected the next day for the second time with fresh retroviral supernatant and incubated overnight. Fresh cRPMI and inhibitors were added every 2–3 days to expand cells. CAR-T cells were debeaded and washed at day 7 for phenotyping and experiments.

### Sarcoma TIL studies.

Sarcoma TIL cultures underwent REP as described (49). Frozen post-REP TIL were thawed and washed with TIL complete media (TIL-CM) consisting of RPMI-1640, 2 mM L–glutamine (HyClone, Thermo Fisher Scientific), 10% heat-inactivated human AB serum (Omega Scientific), 55 μM 2-mercaptoethanol (Invitrogen), 50 μg/mL gentamicin (Invitrogen), 100 IU/mL penicillin, 100 μg/mL streptomycin, and 10 mM HEPES Buffer (Mediatech). Cells were then centrifuged at 300*g* for 5 minutes at room temperature, counted, and resuspended in TIL-CM containing 3,000 IU/mL IL-2 at a concentration of 0.5 × 10^6^ cells/mL. In total, 2 mL of TIL were then cultured/well in 24-well plates. After resting in IL-2 for 24 hours, cells were harvested, counted, and plated at 1 × 10^5^ cells/200 μL in 96-well plates in TIL-CM with 3,000 IU/mL IL-2. Cells were activated using CD3/CD28 Dynabeads (Dynabeads Human T-Activator CD3/CD28, Thermo Fisher Scientific, 11131D) at a 1:1 bead/cell ratio.

### Flow cytometry.

Antibodies (from BioLegend, Tonbo Biosciences, or Becton Dickinson; Supplemental Table 1) were added at 0.2 μg per 1 × 10^6^ cells with 1 μL of FC block per sample in 100 μL volume FACs buffer (PBS, Thermo Fisher Scientific) with 2 mM EDTA (MilliporeSigma), 1% FBS (GeminiBio), and 1% BSA (MilliporeSigma) for 20–30 minutes at 4°C in the dark for surface staining. For CAR-T cell surface staining, 0.5 μL of biotinylated anti-fmc63 per sample in 100 μL volume FACS buffer was added to the cell-surface staining cocktail; then, cells were washed and subsequently stained with 0.2 μL of streptavidin antibody per sample in 100 μL volume FACS buffer for 15 minutes. For intracellular staining, cells stained for surface antigen as described above and washed; then, 100 μL of BD Cytofix/Cytoperm (BD Biosciences, 554722) was added to each tube. Cells were incubated in BD Cytofix/Cytoperm at 4°C for 20 minutes. Afterward, cells were washed twice in 1× BD Perm/Wash buffer (BD Biosciences, 554723), and then, intracellular staining was performed with 0.2 μg per 1 × 10^6^ cells with 1 μL of FC block per sample in 100 μL volume 1× BD Perm/Wash buffer for 1 hour. Viability was determined by staining cells with Ghost Dye Red 780 (Tonbo Biosciences, 50-105-2988) for fixed cells or with DAPI for freshly isolated cells. DAPI staining was performed after surface staining, and Ghost Dye Red 780 was performed before adding surface antibodies. Full antibody information is provided in Supplemental Table 1. All data collection was performed on an LSRII (BD Biosciences), and analysis was performed on FlowJo software (BD Biosciences).

### BM ACT models.

For the long-term BM ACT model, CD8^+^ T cells were isolated from the spleens of CD45.1^+^ OT-I mice by negative magnetic selection using a CD8^+^ T cell kit (Miltenyi Biotec), as described above, and were activated using 0.1 nM SIINFEKL OVA peptide (OVA residues 257–264; Anaspec, AS-60193-1) and 10 ng/mL IL-2 for 48 hours. In total, 2 × 10^6^ activated CD45.1^+^CD8^+^ OT-I T cells were then adoptively transferred into sublethally irradiated (600 RAD; JL Shepherd Mark 1, Model 68A CS-137 Irradiator) 8-week-old CD45.2^+^ C57BL/6J recipient mice (The Jackson Laboratory) by i.v. tail vein injection. On the day of ACT, recipient mice received 10 μg LPS (MilliporeSigma, L5293) in PBS by i.p. injection (40). One month after ACT, recipient mice were vaccinated with 100 μg SIINFEKL peptide and 10 μg LPS in PBS via i.p. injection. BM and spleens were harvested, processed, and analyzed by flow cytometry 60 days after ACT. For the short-term BM ACT model, OT-I activation, mice irradiation, ACT, and LPS injection were performed exactly as described above in the long-term ACT model; however, here, the BM and spleens were harvested and analyzed 7 days after ACT.

### Mouse IFN-γ ELISA.

For cytokine analyses, supernatants were harvested at 48 hours. IFN-γ was quantified from standard curves by ELISA according to the manufacturer’s protocols (LEGEND MAX Mouse IFN-γ ELISA Kit, 430107).

### RNA preparation and qPCR analysis.

Cells pellets were washed with PBS and resuspended and stored in RLT Buffer (for Qiagen kit use) and kept at –80°C until RNA isolation. RNA was isolated following manufacturer’s protocol (Qiagen, 74134; Machery-Nagel, 740955.250) and was quantified immediately or kept at –80°C until quantification. RNA was converted into cDNA per the manufacturer’s protocol (Bio-Rad), and 1–2 ng cDNA was used to quantify the expression of the target genes. The expression of *B2m* and *Ubiquitin* (*Ubc*) transcripts was used to normalize gene expression. Full manufacturer information for all products mentioned throughout Methods is provided in Supplemental Tables 2 and 3. SYBR green (IDT Technologies) probe sequences are provided in Supplemental Table 4.

### Polyamine quantification.

Quantifying polyamine levels was performed as described (50). To extract metabolites, cell pellets (1 × 10^6^ cells per replicate, *n* = 4) were spiked with 5 ng of internal standard solution containing ^13^C_6_-arginine (CLM-2265-H-PK); ^13^C_4_-putrescine (CLM-6574); ^13^C_5_-ornithine (CLM-4724); 1,1,2,2,3,3,4,4-D_8_-N-(3-aminopropyl) butane-1,4-diamine:3HCl (D_8_-spermidine, DLM-9261); or 1,1,2,2,3,3,4,4-D_8_-N,N’-bis(3-aminopropyl)-1,4-butanediamine:4HCl (D_8_-spermine, DLM-9262). All stable isotope-labeled standards were purchased from Cambridge Isotope Labs. A 200 μL aliquot of chilled high-performance liquid chromatography (HPLC) grade methanol (AH365-4, Burdick & Jackson, Honeywell) was added to each sample, vortexed, and incubated for 5 minutes at –80°C. Samples were centrifuged at 16,200*g* for 15 minutes at 4°C. The extraction process was repeated using 100 μL of chilled methanol with incubation at –80°C. The supernatants containing metabolites from both extractions were pooled and dried in a vacuum centrifuge (Savant SC210A SpeedVac Concentrator, Thermo Fisher Scientific) at room temperature for approximately 2 hours. Samples were resuspended in 50 μL of HPLC grade water (Burdick & Jackson, Honeywell).

Liquid chromatography and selected ion monitoring (LC-SIM) was performed on an ultra-performance liquid chromatograph (UPLC model U3000, Dionex) interfaced with an electrospray Q Exactive HF mass spectrometer (Thermo Fisher Scientific) using full MS and LC-SIM for quantification of each target. The following solvent system was used: Solvent A was 100% HPLC grade water (Burdick & Jackson, Honeywell) containing 0.05% heptafluorobutyric acid (LC6206, HFBA, Proteomics Grade, ProteoChem), and solvent B was aqueous 90% acetonitrile (AH015-4, Burdick & Jackson, Honeywell) with 0.1% formic acid (28905, Thermo Fisher Scientific). For each sample, a 10 μL aliquot of the metabolite mixture was loaded onto an Accucore reverse phase C18 column (27826-153030, 2.1 mm × 100 mm, 2.6 μm particle size, Thermo Fisher Scientific). A gradient of 20% B to 80% B was applied over 6 minutes with a flow rate of 0.350 mL/min followed by reequilibration over 3 minutes, for a total of 9 minutes for the LC experiment. Mass spectrometry (MS) instrument parameters for SIM on the Q Exactive HF included the following: resolution, 70,000; isolation window width, 1.0 *m/z* and isolation offset 0.2 *m/z*; AGC target, 2 × 10^5^; maximum injection time (IT), 100 ms; and an inclusion list containing the *m/z* for each metabolite, its respective stable isotope standard, and a scheduled time window for metabolite elution.

Data analysis for quantifying metabolites used the Xcalibur Quan Browser (version 3.0.63, Thermo Fisher Scientific). Metabolite amounts (ng) were calculated using the peak area ratio of each molecule to its respective stable isotope standard (SIS). The relative amount of glutamine was determined using arginine isotope-labeled internal standard.

### Metabolite tracing.

Activated OT-I T cells were washed in DMEM containing high glucose and no arginine, glutamine, or lysine (14431, Thermo Fisher). In total, 1 × 10^6^ cells were then labeled with either 2 mM ^13^C-arginine (Cambridge Isotopes, fully labeled) and 2 mM ^12^C-glutamine (Thermo Fisher Scientific) or 2 mM ^13^C-glutamine (Cambridge Isotopes, fully labeled) and 2 mM ^12^C-arginine (Sigma Aldrich) for 3 or 6 hours at 37°C, at which time media was removed from the cells and pellets were immediately placed on ice.

For metabolite extraction, 500 μL of prechilled cold methanol was added to each cell pellet, and the sample was vortexed and incubated at –80°C for 20 minutes. Samples were centrifuged at 14,000*g* for 5 minutes at 4°C, and the metabolite extract was collected in a clean microcentrifuge tube. The pellet was reextracted as described above and the metabolite extracts were pooled. Metabolite extracts were then dried in a vacuum centrifuge (Savant SC210A SpeedVac Concentrator, Thermo Fisher Scientific) and resuspended in LC loading solvent.

Isotope tracing with LC–high-resolution MS was used for targeted isotope tracer analysis. In total, 5 μL of each sample was analyzed using an ultra-high performance liquid chromatograph (Vanquish, Thermo Fisher Scientific) interfaced with a Q Exactive HF mass spectrometer (Thermo Fisher Scientific) using full scan mode. Samples were separated using an Accucore reversed phase C18 column (27826-153030, 2.1 mm ID × 100 mm length, 2.6 μm particle size, Thermo Fisher Scientific) at 350 μL/min. A gradient starting at 20% B was held for 2 minutes and was then ramped from 20% B to 50% B from 2 to 4 minutes and from 50% B to 80% B from 4 to 6 minutes, followed by reequilibration over 3 minutes, for a total of 9 minutes for each experiment. Solvent A was 100% HPLC grade water (LC365-4, Burdick & Jackson, Honeywell) containing 0.05% Heptafluorobutyric acid (LC6206, HFBA, Proteomics Grade, ProteoChem), and solvent B was 100% HPLC grade methanol (LC230-4, Burdick & Jackson, Honeywell) containing 0.05% Heptafluorobutyric acid (LC6206, HFBA, Proteomics Grade, ProteoChem); HFBA was used as counter ion to enable analysis of the polyamines. Samples were analyzed in positive ion mode using an ESI voltage of 3.5 kV. MS instrument parameters for SIM included the following: resolution, 70,000; AGC target, 3 × 10^6^; maximum IT, 100 ms.

Xcalibur Quan Browser (version 3.0.63, Thermo Fisher Scientific) was used to extract peak heights from the chromatograms for the six ^13^C-labeled and unlabeled endogenous metabolites: arginine, glutamine, putrescine, ornithine, spermine, and spermidine. Apparent fractional labeling was calculated by determining how many ^13^C molecules were present versus the total (^12^C + ^13^C) for each metabolite.

### ^14^C-arginine and ^14^C-glutamine uptake assays.

Uptake assays were performed using activated (SIINFEKL peptide stimulated) and nonactivated (Trp2 peptide stimulated) CD8^+^ OT-I T cells at 24 hours. In total, 3 × 10^5^ cells per replicate were suspended in 1× PBS with Ca^2+^ and Mg^2+^ (Thermo Fisher Scientific) containing 0.1% glucose (MilliporeSigma, G8270). Samples were incubated in triplicate with either 0.1 μCi L-[^14^C(U)]-glutamine (Perkin Elmer, #NEC451050UC), L-[^14^C(U)]-arginine (Perkin Elmer, NEC267E050UC), L-[1-^14^C]-ornithine (Perkin Elmer, NEC710250UC), or L-[methyl-^3^H]-methionine (Perkin Elmer, NET061X) and incubated at room temperature for 30–60 minutes. Cells were washed twice with ice cold 1× PBS, and cell pellets were dissolved in 400 μL 25 mM NaOH and measured in 5.6 mL Optiphase Hisafe 2 (Perkin Elmer, 1200-436) using a TriCarb liquid scintillation analyzer (Perkin Elmer).

### ODC enzyme assay.

CD8^+^ OT-I T cells were activated for 24 hours; then, 2 × 10^6^ cells per replicate were harvested and washed with PBS. In total, 0.5 mL of ODC breaking buffer (25 mM Tris-Cl [pH 7.5], 0.1 mM EDTA, and freshly made 2.5 mM DTT) was added, and cells were then frozen immediately in a dry ice/ethanol bath. Afterward, cells underwent 3 freeze/thaw cycles and were then centrifuged at 15,871*g* for 5 minutes at room temperature. A blunt needle 19 gauge, 1.5 inch (Covidien Monoject, 8881202355) was assembled on a filter (EMD Millipore, AP2501000) fitted on a flow tube cap. Then, 45 μL of Soluene 350 was added on the filter. In total, 1 μL of ^14^C-ornithine was then added to the bottom of the flow tube (250 μCi/2.5 mL = 0.1 μCi/μL) and the 200 μL of the T cell lysate was added. The flow tube was capped and incubated at 37°C for 1 hour. In total, 200 μL of 50% TCA was then added, and the cell lysates were incubated at ambient temperature for 30 minutes. The filter was then taken to count disintegrations per minute (DPM). Values were normalized to cell number.

### Statistics.

Data were analyzed using GraphPad Prism 8. A 2-tailed *t* test was used to analyze experiments with 2 groups. *P* <0.05 was considered statistically significant. Two-way or 1-way ANOVA with appropriate post hoc tests were used as indicated in the figure legends. For comparison of the experimental groups with the Ctrl, we used Dunnett’s post hoc test, and for comparing experimental groups among each other, we used Tukey’s post hoc test. Data are represented as mean ± SEM or SD, as indicated in figure legends. The numbers of animals in each cohort are listed in the figure legends for experiments entailing biological replicates. Biological replicate experiments are representative of 2–3 independent experiments and contain 3 technical replicates unless otherwise stated in the figure legends. ACT studies with vehicle control and DFMO- or GC7-pretreated CD45.1^+^CD8^+^ OT-I cells (Figures 6 and 7) were performed in 5 CD45.2^+^ recipient mice for each cohort. Technical replicate experiments contain 3 technical replicates. Group sizes of cohorts were based on prior publications with similar studies. The graphical abstract was generated using BioRender.

### Study approval.

Human TIL harvest and collection were performed under the IRB-approved protocol MCC 18609 entitled “Use of Sarcoma Tumor Specimens Not Required for Diagnostic Purposes, and Peripheral Blood for Validation and Characterization of Tumor Infiltrating Lymphocyte (TIL) Growth Procedures” (IRB ID Pro00025312_CR000004). All experiments were approved by the IACUC of the Moffitt Cancer Center and the University of South Florida.

### Data availability.

All data that are presented in the figures associated with the main text or that are presented in the supplemental figures are provided in the Supporting Data Values file.

## Author contributions

AGE and RSH designed and performed the experiments, analyzed the data, and prepared the figures. AGE, PKEB, and JLC conceived the study and designed experiments. CY performed ODC enzyme assays, and MRF performed ^14^C-arginine, ^14^C-glutamine, ^14^C-ornithine, and L-[methyl-^3^H]-methionine uptake assays. OP provided advice and the PTI. LNFD, ML, and JMK performed MS analyses of polyamines and the ^13^C isotope tracer analysis. AGE performed and analyzed the in vitro T cell experiments, the ACT studies, and the flow cytometry experiments. JEM and SAPT provided advice and the pre- and post-REP human TIL from sarcoma TIL studies. RA manufactured the human CAR-T cells, and RA and FLL provided advice on CAR-T cell studies. JLC supervised the work and obtained funding. AGE and JLC wrote the manuscript. All authors reviewed and edited the manuscript.

## Supplementary Material

Supplemental data

Supporting data values

## Version 1 08/15/2023

In-Press Preview

## Version 2 09/22/2023

Electronic publication
