# Supplementary material for: Modulating the polyamine/hypusine axis controls generation of CD8+ tissue-resident memory T cells
Source: JCI Insight. 2023 Sep 22;8(18):e169308. doi: 10.1172/jci.insight.169308 (PMC10561731; doi:10.1172/jci.insight.169308)
Supplement: Supplemental data [file jciinsight-8-169308-s159.pdf]

Supplemental Figure 1

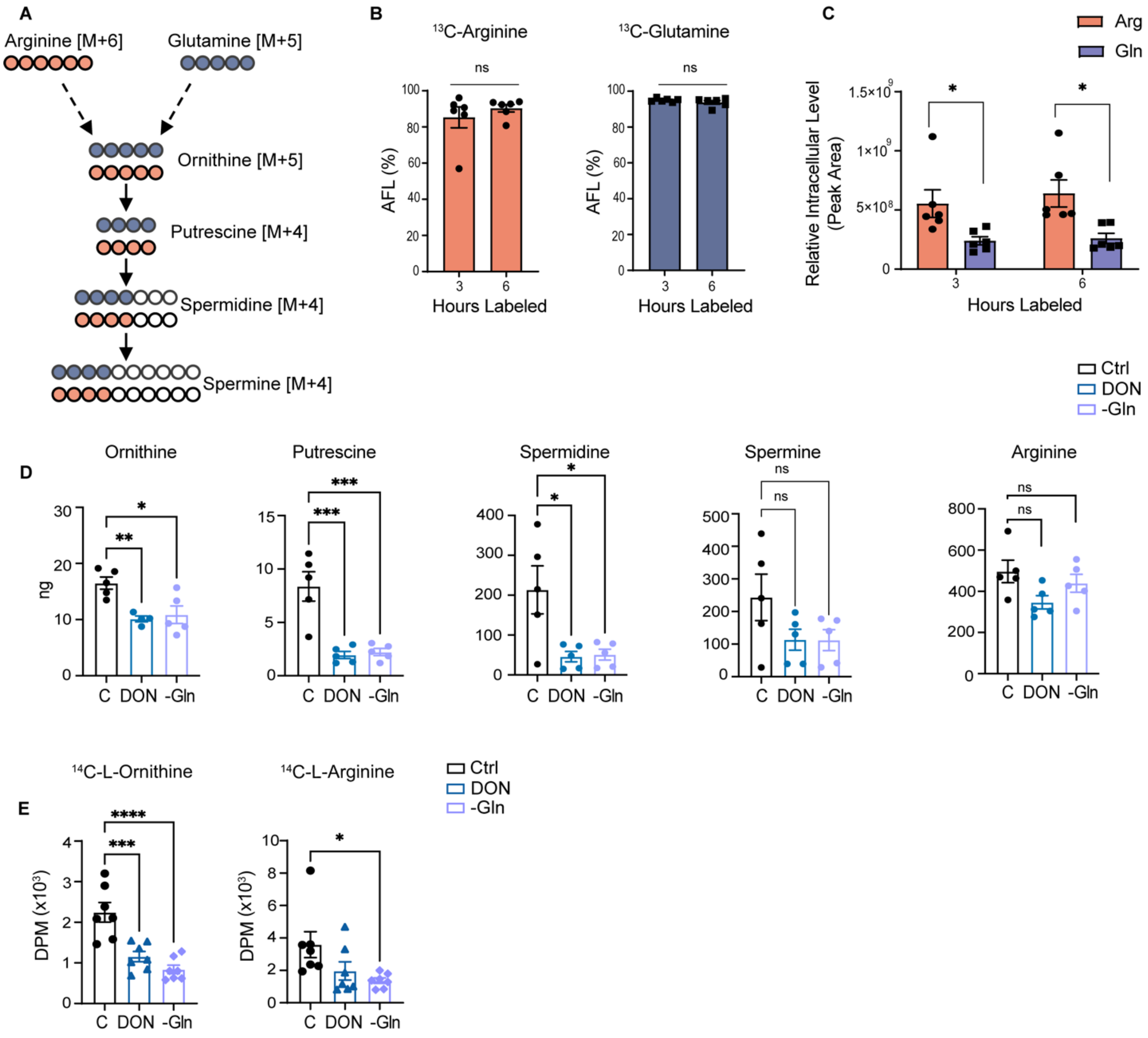

Supplemental Figure 1. Glutamine catabolism primarily drives polyamine biosynthesis in antigen activated CD8⁺ T cells. (**A**) Isotope labeling strategy assessing the production of polyamines from glutamine or arginine in antigen-activated mouse OT-I CD8⁺ T cells. (**B**) Complete labeling of CD8⁺ T cells with ^{13}C-glutamine or ^{13}C-arginine (*n* = 6). (**C**) Relative intracellular levels (peak area) of ^{13}C-glutamine or ^{13}C-arginine at 3 and 6 hours of labeling (*n* = 6). (**D**) Levels of intracellular ornithine, putrescine, spermidine, spermine and arginine were determined by liquid chromatography-mass spectrometry in OT-I T cells activated in the presence of DON or in glutamine deficient media (*n* = 5). Uptake of (**E**) ^{14}C-L-ornithine and ^{14}C-L-arginine in OT-I T cells activated in the presence of DON or in glutamine deficient media (*n* = 7). Data in (**B**) were analyzed by unpaired t-test and data in (**C**) were analyzed two-way ANOVA. Data in (**D**,**E**) were analyzed using one-way ANOVA and Dunnett multiple comparisons. Each dot represents a biologically independent replicate and data are mean ± SEM (*, $P < 0.05$; **, $P < 0.01$; ***, $P < 0.001$; ****, $P < 0.0001$).

Supplemental Figure 2

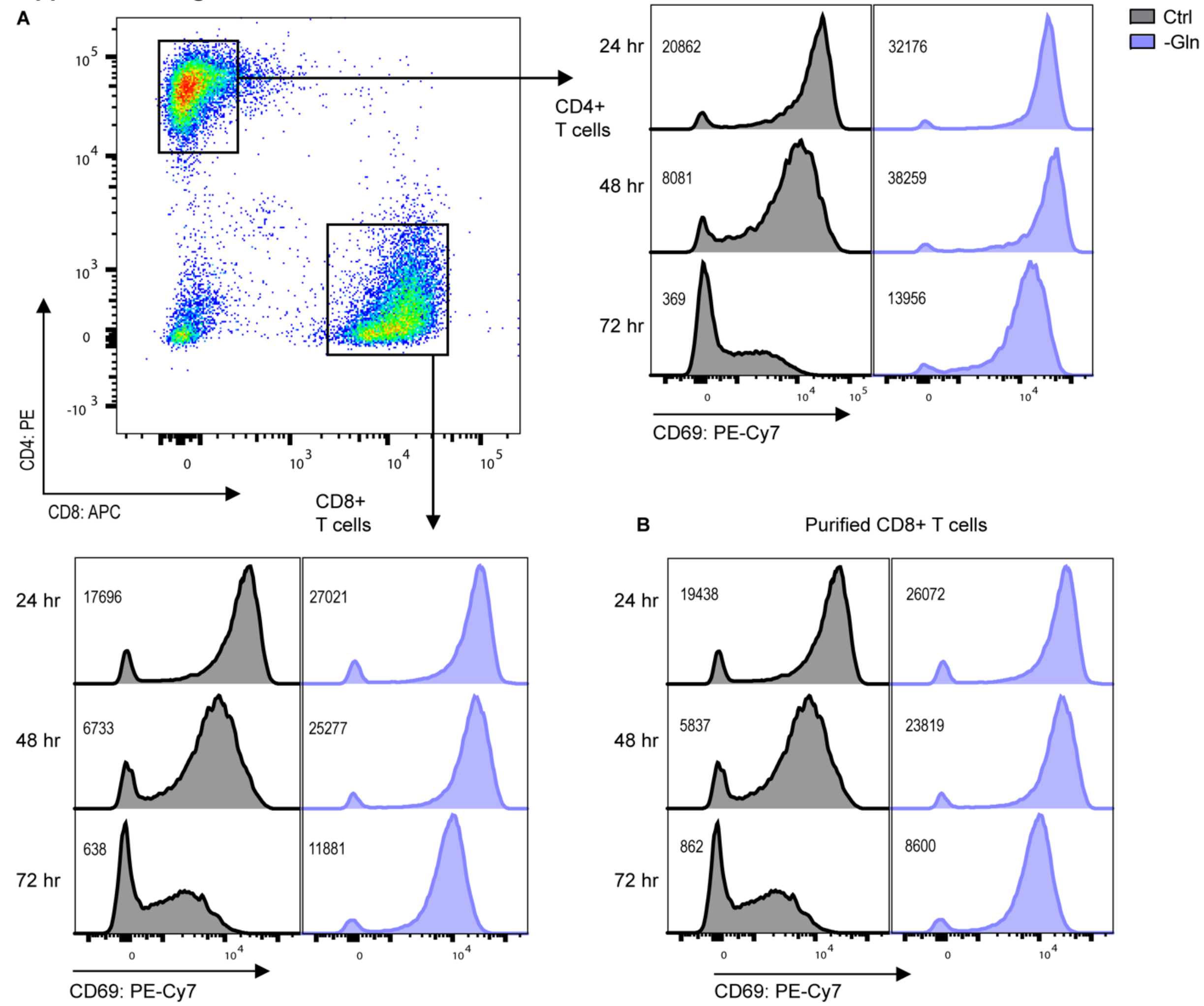

Supplemental Figure 2. Representative histograms of CD69 expression in activated polyclonal T cells and purified CD8⁺ T cells under glutamine deficient conditions. Representative FACS plots of (**A**) polyclonal CD4⁺ and CD8⁺ T cells, and (**B**) purified CD8⁺ T cells, at 24-, 48- and 72-hours post-activation, when cultured in glutamine-replete (Ctrl) *vs.* glutamine-deficient (-Gln) media.

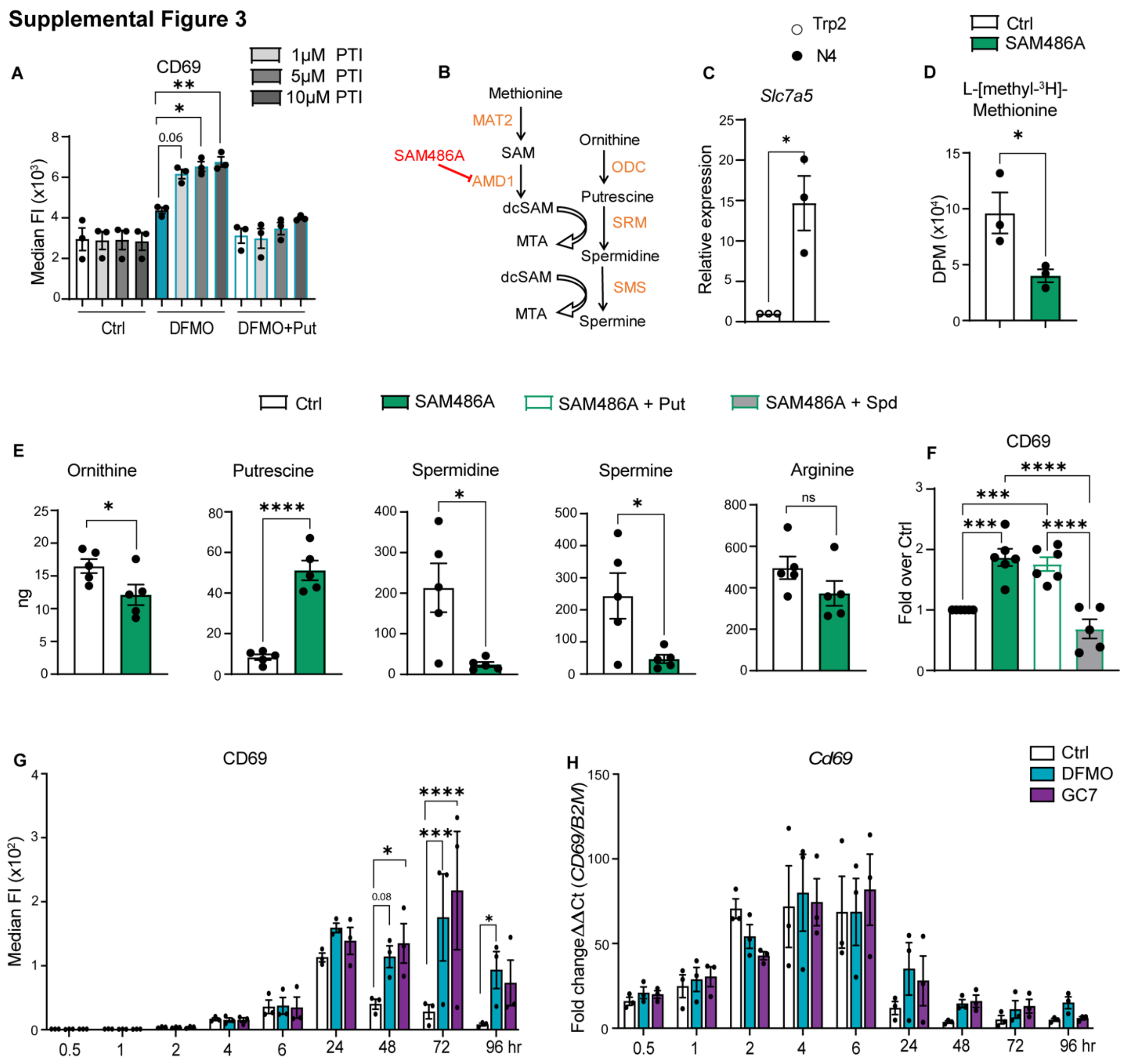

Supplemental Figure 3. Polyamine transport inhibitor augments DFMO-induced increases in CD69 and effects of DFMO and GC7 treatment on CD69 cell surface *vs.* mRNA levels. (**A**) CD69 median fluorescence (FI) in OT-I CD8$^+$ T cells activated without (Ctrl) or with added DFMO (5 mM) ± Put (500 µM) and increasing concentrations (0, 1, 5 and 10 µM) of the polyamine transport inhibitor (PTI) Trimer44NMe (n = 3). (**B**) Schematic of metabolic pathway connecting methionine to the polyamines. (**C**) Fold change in levels of *Slc7a5* mRNA (determined by qRT-PCR) normalized to *B2m* mRNA (n = 3). (**D**) Uptake of L-[methy-^{3}H]-methionine as DPM (n = 3). (**E**) Intracellular levels of ornithine, putrescine, spermidine, spermine and arginine were determined by LC-MS in OT-I T cells activated +/- 10 µM SAM486A (n = 5). (**F**) Fold over Ctrl of median FI of CD69 in OT-I T cells activated in the presence of 10 µM SAM486A +/- 500 µM Put or 100 µM Spd (n = 6). (**G**, **H**) Polyclonal CD8$^+$ T cells T cells were activated without (Ctrl) or with added DFMO (5 mM) or GC-7 (10 µM) (n = 3). At the indicated intervals, cells were harvested and analyzed for (**G**) CD69 cell surface expression by flow cytometry or (**H**) *CD69* mRNA levels by qRT-PCR. *CD69* mRNA expression was normalized to levels of *B2m* mRNA. Data in (**A**) were analyzed using one-way ANOVA with Tukey's post-hoc test. Data in (**C**, **E**) were analyzed using unpaired t-test and data in (**F**) were analyzed using one-way

ANOVA with Dunnett multiple comparisons. Data in (**G**, **H**) were analyzed using two-way ANOVA and Dunnett multiple comparisons. Each dot represents a biological replicate, and all data are mean $\pm$ SEM, (*, $P < 0.05$; **, $P < 0.01$; ***, $P < 0.001$; ****, $P < 0.0001$).

Supplemental Figure 4

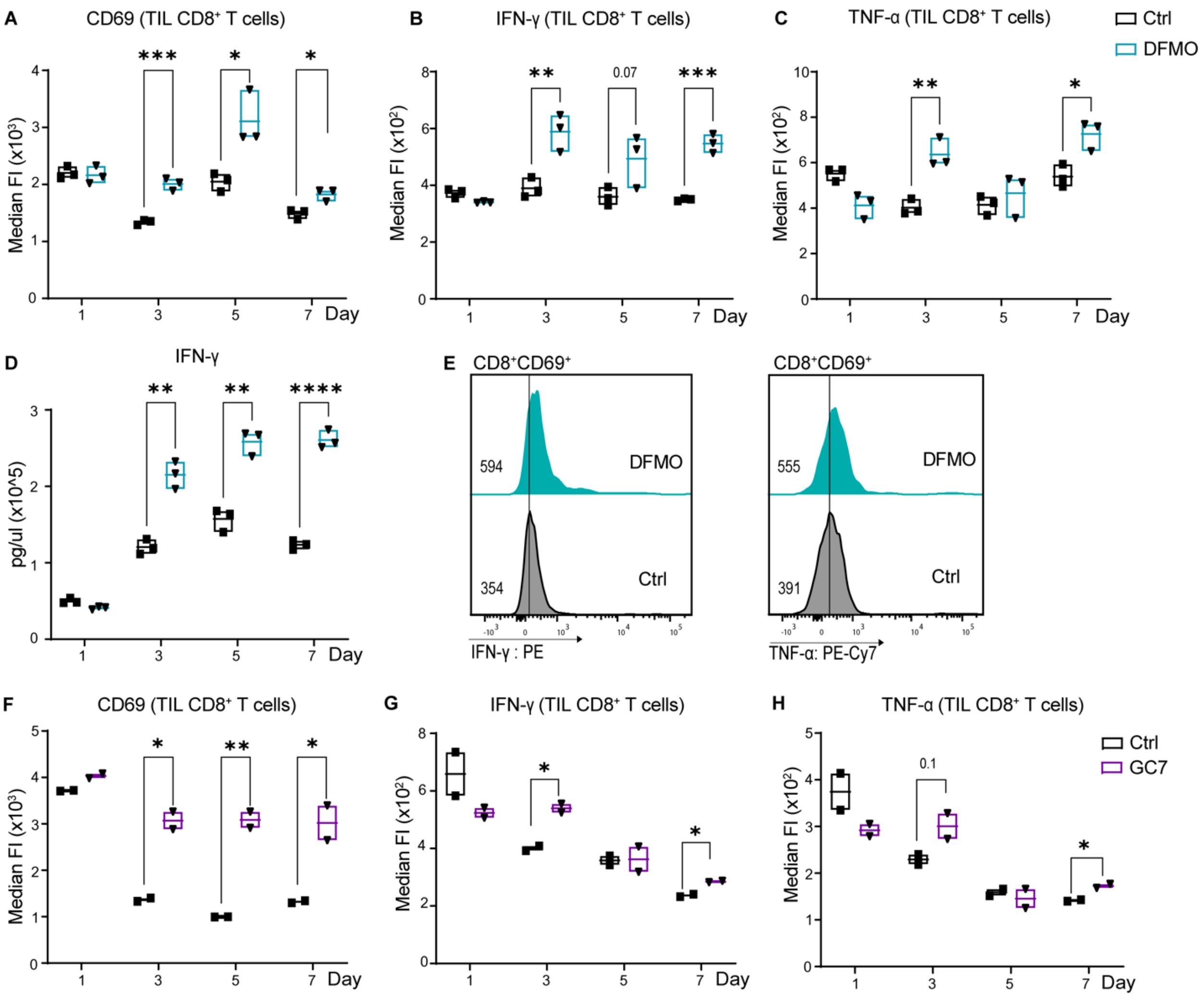

Supplemental Figure 4. Inhibition of the polyamine-hypusine axis controls CD69 expression and IFN-γ **and TNF-α production in human sarcoma TIL $CD8^+$ T cells.** Median FI of (**A**) CD69, (**B**) IFN-γ and (**C**) TNF-α in anti-CD3/CD28-activated sarcoma TIL $CD8^+$ T cells treated without (Ctrl) or with 5 mM DFMO for the indicated intervals. (**D**) IFN-γ ELISA from human sarcoma activated TIL $CD8^+$ T cells (Ctrl) or TIL treated with 5 mM DFMO for the indicated intervals. (**E**) Flow plots of IFN-γ (*left*) and TNF-α (*right*) levels from activated $CD69^+$ $CD8^+$ TILs (Ctrl) or TIL treated with 5 mM DFMO. Median FI of (**F**) CD69, (**G**) IFN-γ (*left*) and (**H**) TNF-α in activated TIL $CD8^+$ T cells (Ctrl) or TIL treated with 10 µM GC7 for the indicated intervals. All data are representative of 3 independent experiments and were analyzed using multiple t-tests and Holm-Šídák test. All data are mean ± SD, (*, $P < 0.05$; **, $P < 0.01$; ***, $P < 0.001$; ****, $P < 0.0001$).

Supplemental Figure 5

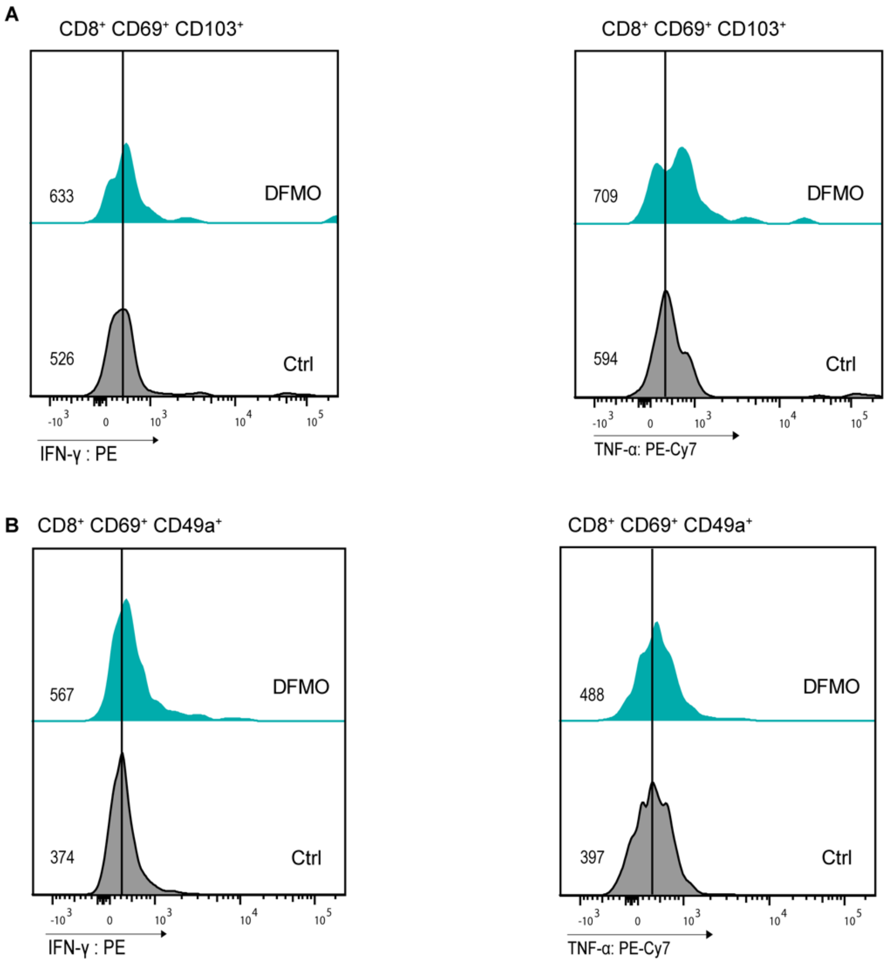

Supplemental Figure 5. Blocking polyamine biosynthesis promotes the differentiation of T_{RM}-like cells ex vivo. (**A**) Flow cytometry analyses of levels of IFN-γ (*left*) and TNF-α (*right*) in TGF-β-treated, anti-CD3/CD28 activated mouse $CD8^+$ T cells (Ctrl) and in these cells treated with DFMO (5 mM). Levels of IFN-γ and TNF-α were determined in $CD69^+CD103^+$ $CD8^+$ T cells. (**B**) Flow cytometry analyses of levels of IFN-γ and TNF-α in TGF-β-treated, anti-CD3/CD28 activated human sarcoma $CD8^+$ TIL (Ctrl) and in these cells treated with DFMO (5 mM). Levels of IFN-γ and TNF-α were determined in $CD69^+CD49a^+$ $CD8^+$ T cells.

Supplemental Figure 6

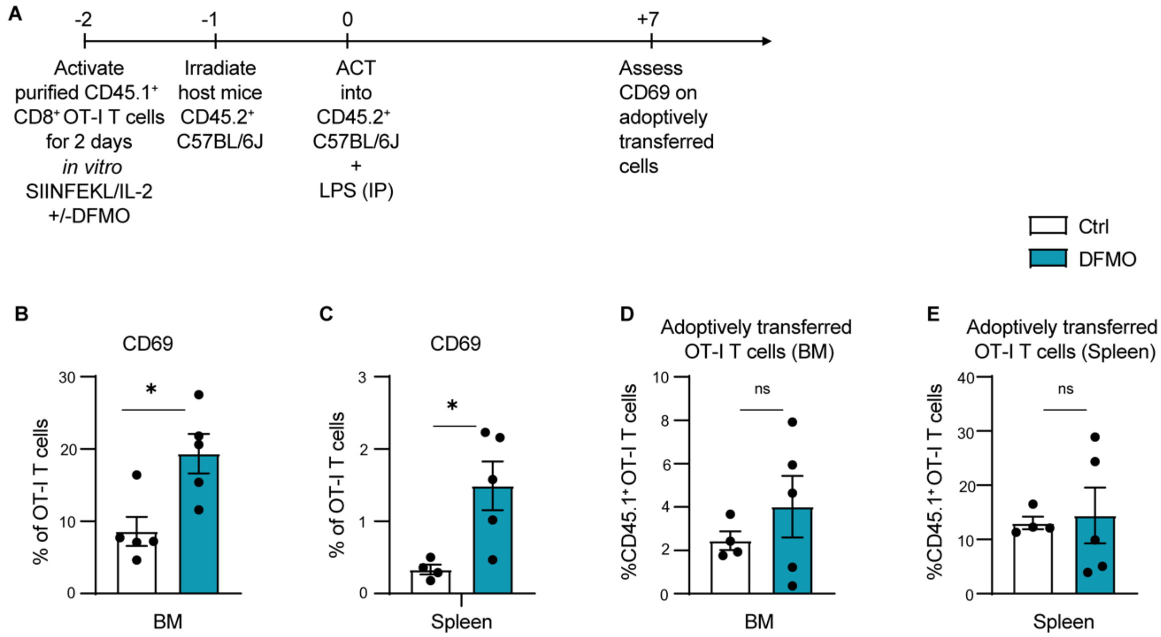

Supplemental Figure 6. Inhibition of polyamine biosynthesis augments generation of bone marrow T_{RM} after short-term ACT. (**A**) Schematic of the experimental design of the short-term bone marrow T cell adoptive cell transfer (ACT) model. (**B**, **C**) After 7 days post ACT, mice were assessed for percentage of CD69$^+$ of CD45.1$^+$CD8$^+$ OT-I T cells in the bone marrow (**B**) and spleen (**C**) of CD45.2$^+$ recipient mice (n = 5). (**D**, **E**) Percentage of CD45.1$^+$CD8$^+$ OT-I T cells in the bone marrow (**D**) and spleen (**E**) of CD45.2$^+$ recipient mice (n = 5). All data were analyzed using unpaired t-test. Each dot represents a biological replicate, and all data are mean ± SEM, (*, $P < 0.05$).

Supplemental Table 1. Flow antibodies, dyes and reagents.

Reagent	Source	Identifier
CD69, mouse, PE-CF594, PE-Cy7 or APC	Tonbo, Biolegend or BD	60-0691, 104514 or 562455<br>Clone: H1.2F3
CD8α, mouse, BV711, FITC or BUV395	Biolegend, Tonbo or BD	100759, 35-1886, 565968<br>Clone:53-6.7
CD4, mouse, BV510	Biolegend	100553<br>Clone: RM4-5
CD103, mouse, BV785, PE or BV421	Biolegend, BD	121439, 557495, 562771<br>Clone:2E7, M290
Ly6C, mouse, APC	Biolegend	128016<br>Clone:HK1.4
CXCR6, mouse, PE/Dazzele	Biolegend	151117<br>Clone:SA051D1
CD45.1, mouse, PerCP/Cy5.5	Biolegend	110728<br>Clone:A20
Bcl-2, mouse, AF647	Biolegend	633510<br>Clone: BCL/10C4
CD8α, human, FITC, BUV737 or BV711	Tonbo, BD or Biolegend	35-0089, 612755, 301044<br>Clone:hit8a, SK1, RPA-T8
CD69, human, BV605, APC/Cy7 or FITC	Biolegend	310938, 310904, 310914<br>Clone:FN50
CD3, human, BV711	Biolegend	317328<br>Clone:OKT3
CD103, human, APC	BD	563883<br>Clone:Ber-ACT8
CD49a, human, APC/Fire750	Biolegend	328318<br>Clone:TS2/7
IFN-γ, human, PE	BD	554701
TNF-α, human, PE-Cy7	Biolegend	502930<br>Clone:MAb11
BCL2, human, AF647	Biolegend	563600<br>Clone:Bcl-2/100
Granzyme B, human, AF700	Biolegend	372222<br>Clone:QA16A02
Perforin, human, BV510	Biolegend	308120<br>Clone:dG9
DAPI (4',6-Diamidino-2-Phenylindole, Dihydrochloride)	Thermo Fisher	#D1306
Ghost Dye Red 780	Tonbo	#13-0865-T500
Brefeldin A	Invitrogen	#4506-51
BD Cytofix/Cytoperm™	BD	#554714

Supplemental Table 2. Key resources.

Reagent	Source	Identifier
OVA (257-264)	Anaspec	#AS-60193-1
Trp2 (180-188)	Anaspec	#AS-61058
Recombinant Murine IL-2	Peprotech	#212-12
13C5-glutamine	Cambridge Isotope Labs	# CLM-1822-H
^{13}C6-arginine	Cambridge Isotope Labs	# CLM-2265-H
^{13}C5-L-proline	Cambridge Isotope Labs	# CLM-2260-H-PK
^{13}C5-L-methionine	Cambridge Isotope Labs	# CNLM-759-H-PK
L-[^{14}C(U)]-arginine	Perkin Elmer	#NEC267E050UC
L-[^{14}C(U)]-glutamine	Perkin Elmer	#NEC451050UC
L-[1-^{14}C]-ornithine	Perkin Elmer	#NEC710250UC
L-[methyl-^{3}H]-methionine	Perkin Elmer	#NET061X
dimemethyl-α-ketoglutarate (DMα-KG)	TCI America	13192-04-6 #K0013
L-ornithine monohydrochloride	Sigma	#O2375
Putrescine	Sigma	#P5780
Spermidine	Sigma	#S2626
Spermine	Sigma	#S3256
Dimethyl DL-glutamate hydrochloride	TCI America	13515-99-6 #D3305
difluoromethylornithine (DFMO)	Dr. Patrick M. Woster	Medical University of South Carolina
GC7	Sigma	#259545
Nω-hydroxy-nor-L-arginine (nor-NOHA)	Sigma	#189302-40-7
6-Diazo-5-oxo-L-norleucine (DON)	Sigma	#D2141-5MG
SAM486A or sardomozide	MedChemExpress	#HY-13746B
rhTGF-β1	R and D Systems	#240-B/CF
rmTGF-β1	R and D Systems	#7666-MB
Murine IL-2	Peprotech	#212-12
Human IL-2	Peprotech	#200-02
Dynabeads™ Mouse T-Activator CD3/CD28	Gibco	11453D
Dynabeads™ Human T-Activator CD3/CD28	Gibco	11131D
Power SYBR™ Green PCR Master Mix	Thermo Fisher	#4367659
Lipopolysaccharide (LPS) from *E. coli* O111:B4	Sigma	L5293
RPMI 1640 Medium	Thermo Fisher	#11875
[-Gln] RPMI 1640 Medium	Thermo Fisher	#21870-076
SILAC DMEM flex media	Thermo Fisher	#A2493901

Supplemental Table 3. Human samples and kits.

Human samples	Source	Identifier
Human PBMCs	One Blood, St Petersburg, FL<br>Lifesouth community blood centers	NA
**Kits**	**Source**	**Identifier**
LEGEND MAX™ Mouse IFN-γ ELISA Kit	Biolegend	#430107
Pan T cell Isolation Kit II, mouse	Miltenyi	#130-095-130
CD8a$^+$ T cell Isolation Kit, mouse	Miltenyi	#130-104-075
CD8a$^+$ T cell Isolation Kit, human	Miltenyi	#130-096-495
RNeasy Plus Mini Kit	Qiagen	#74134

Supplemental Table 4. qRT-PCR primers

Gene	Sequence 5'-3'
Slc1a5 Forward	CTGCCTGTGAAGGACATCTCCT
Slc1a5 Reverse	CTCGGCATCTTGGTTCGATCCA
Slc7a1 Forward	TGGTCTTGTGCTTCATCGTG
Slc7a1 Reverse	GACACCAGAGAATCCAAAGGG
Slc38a1 Forward	TTACCAACCATCGCCTTC
Slc38a1 Reverse	ATGAGAATGTCGCCTGTG
Slc38a2 Forward	GGTATCTGAACGGTGACTATCTG
Slc38a2 Reverse	TCTGCGGTGCTATTGAATGC
Slc7A5 Forward	GGTCTCTGTTCACGTCCTCAAG
Slc7A5 Reverse	GAACACCAGTGATGGCACAGGT
B2m Forward	TTCTGGTGCTTGTCTCACTGA
B2m Reverse	CAGTATGTTCGGCTTCCCATTC
Odc1 Forward	GACGAGTTTGACTGCCACATC
Odc1 Reverse	CGCAACATAGAACGCATCCTT
Oat Forward	GGAGTCCACACCTCAGTCG
Oat Reverse	CCACATCCCACATATAAATGCCT
*Aldh18a1*Forward	CGTCATCACAGACATCGTGGAG
Aldh18a1 Reverse	GGCTCTAAGGTAGCCAGCATTC
CD69 Forward	GGGCTGTGTTAATAGTGGTCCTC
CD69 Reverse	CTTGCAGGTAGCAACATGGTGG
